# IGF2BP3 remodels RISC occupancy to control microRNA targeting in leukemia

**DOI:** 10.1093/narcan/zcag025

**Published:** 2026-09-23

**Authors:** Lyna E S Kabbani, Shruti Kapoor, Gunjan Sharma, Martin Gutierrez, Zachary T Neeb, Amit K Jaiswal, Alexander J Ritter, Sol Katzman, Jordyn E Feldman, Dinesh S Rao, Jeremy R Sanford

**Affiliations:** Department of Molecular, Cell and Developmental Biology and Center for Molecular Biology of RNA, University of California Santa Cruz, Santa Cruz, CA 95064, United States; Department of Molecular, Cell and Developmental Biology and Center for Molecular Biology of RNA, University of California Santa Cruz, Santa Cruz, CA 95064, United States; Department of Pathology and Laboratory Medicine, University of California, Los Angeles, CA 90095, United States; Department of Molecular, Cell and Developmental Biology and Center for Molecular Biology of RNA, University of California Santa Cruz, Santa Cruz, CA 95064, United States; Department of Molecular, Cell and Developmental Biology and Center for Molecular Biology of RNA, University of California Santa Cruz, Santa Cruz, CA 95064, United States; Department of Pathology and Laboratory Medicine, University of California, Los Angeles, CA 90095, United States; Department of Biomolecular Engineering, University of California Santa Cruz, Santa Cruz, CA 95064, United States; Center for Biomolecular Science and Engineering, University of California Santa Cruz, Santa Cruz, CA 95064, United States; Department of Molecular, Cell and Developmental Biology and Center for Molecular Biology of RNA, University of California Santa Cruz, Santa Cruz, CA 95064, United States; Department of Pathology and Laboratory Medicine, University of California, Los Angeles, CA 90095, United States; Jonsson Comprehensive Cancer Center, University of California, Los Angeles, CA 90024, United States; Broad Stem Cell Research Center, University of California, Los Angeles, CA 94143, United States; Department of Molecular, Cell and Developmental Biology and Center for Molecular Biology of RNA, University of California Santa Cruz, Santa Cruz, CA 95064, United States; Center for Biomolecular Science and Engineering, University of California Santa Cruz, Santa Cruz, CA 95064, United States

## Abstract

IGF2BP3 is an oncofetal RNA-binding protein that promotes leukemogenesis, but the mechanisms by which it remodels post-transcriptional gene regulation remain incompletely understood. Because IGF2BP3 binds extensively within 3′ untranslated regions and has been implicated in microRNA (miRNA)-mediated regulation, we asked whether IGF2BP3 controls access of the RNA-induced silencing complex (RISC) to endogenous messenger RNA (mRNA) targets in leukemia cells. Using AGO2 miR–eCLIP in control and IGF2BP3-deficient MLL–AF4 B-lymphoblastic leukemia cells, we found that loss of IGF2BP3 broadly redistributes AGO2 occupancy toward 3′ UTRs of IGF2BP3-bound transcripts. AGO2 gains were enriched near miRNA target sites and on transcripts associated with proliferative and oncogenic pathways. Chimeric AGO2-miRNA reads revealed transcript- and miRNA-specific remodeling of RISC interactions, with enhanced miR-181 occupancy on oncogenic transcripts following IGF2BP3 loss. Functionally, miR-181a overexpression impaired leukemic cell growth, partially phenocopying IGF2BP3 deletion. Motif analyses indicated that IGF2BP3 and AGO2 converge on related 3′ UTR sequence environments, while biochemical competition assays demonstrated that purified IGF2BP3 can displace AGO2-containing RISC complexes from a shared RNA substrate. Together, these findings support a model in which IGF2BP3 restricts RISC access to select 3′ UTR regulatory elements, thereby reshaping miRNA–target interactions and sustaining leukemic gene expression programs. Rather than acting solely as an mRNA stabilizing factor, IGF2BP3 functions as a transcript-selective regulator of RISC accessibility in leukemia.

## Graphical Abstract

**Graphical Abstract:**
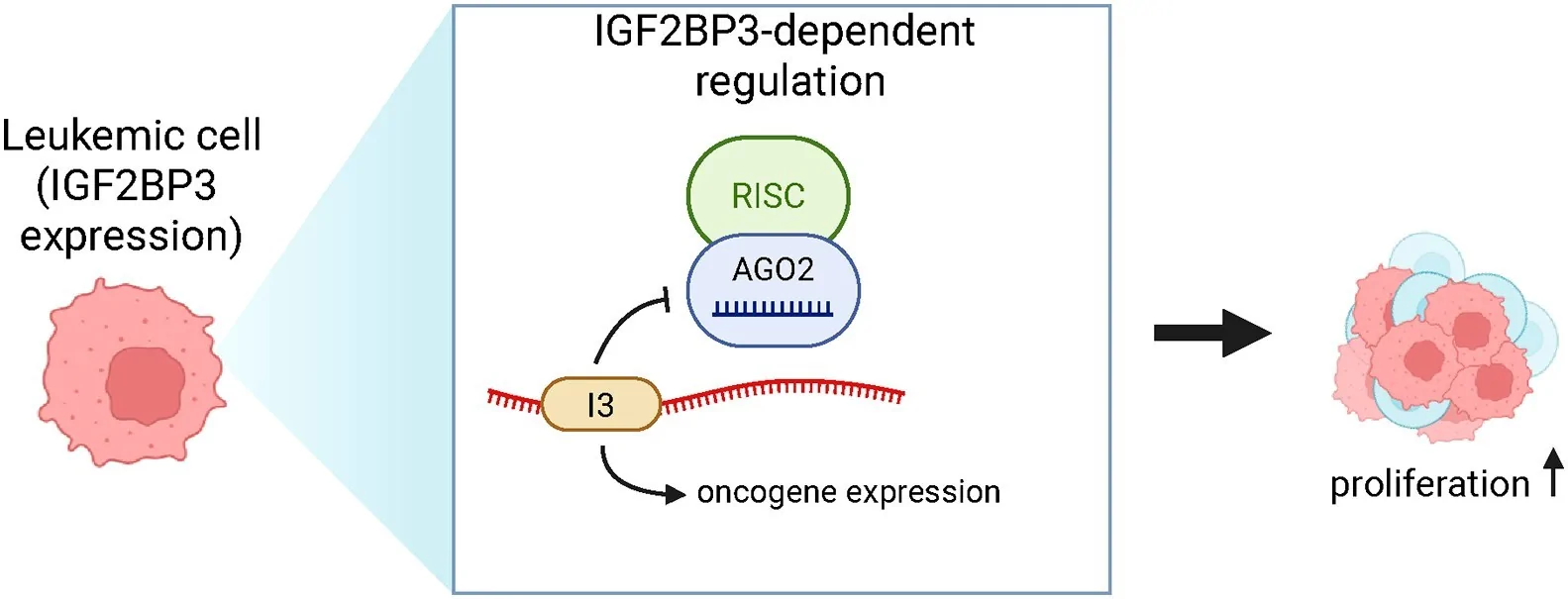

## Introduction

B-cell acute lymphoblastic leukemia (B-ALL) is the most common pediatric leukemia and disproportionately affects children and adolescents, with approximately 3000 new cases diagnosed annually in the United States [1, 2]. B-ALL is characterized by the expansion of immature lymphoid progenitors that disrupt normal hematopoiesis and immune function. Although survival rates for pediatric B-ALL have improved substantially over the past several decades, current treatment regimens remain highly intensive and are associated with significant long-term morbidity, with nearly 90% of survivors experiencing chronic adverse health effects related to chemotherapy and radiation exposure [3–5]. These clinical challenges underscore the need to better understand the molecular mechanisms that drive leukemogenesis and post-transcriptional gene dysregulation in B-ALL.

Insulin-like growth factor 2 mRNA-binding protein 3 (IGF2BP3) is an oncofetal RNA-binding protein (RBP) that is highly expressed during embryogenesis but largely absent from mature tissues, with the exception of select reproductive tissues [6–8]. Aberrant re-expression of IGF2BP3 is observed across multiple malignancies and frequently correlates with disease progression and poor clinical outcome [9]. In hematologic cancers, our group and others have identified IGF2BP3 as a critical regulator of leukemogenesis [10–13]. IGF2BP3 promotes expansion of immature hematopoietic progenitors and is required for the maintenance and function of leukemic stem and progenitor cells *in vivo* [10, 11]. Consistent with these observations, genetic depletion of IGF2BP3 markedly attenuates mixed lineage leukemia (MLL)-driven leukemogenesis in mouse models and sensitizes leukemic cells to targeted therapeutic inhibition [11, 13]. Despite mounting evidence that IGF2BP3 functions as a central driver of oncogenic gene regulation, the molecular mechanisms through which IGF2BP3 remodels post-transcriptional gene expression programs in leukemia remain incompletely understood.

For most messenger RNA (mRNA) transcripts, 3′ untranslated regions (3′ UTRs) harbor numerous *cis*-regulatory elements that govern post-transcriptional gene expression [14–16]. IGF2BP3 preferentially associates with 3′ UTRs and modulates post-transcriptional regulation through effects on mRNA stability, localization, and interactions with regulatory RNA machinery [8, 17, 18]. Previous studies suggested that IGF2BP3 can modulate microRNA (miRNA)-mediated repression, but the molecular basis of this interaction remains unclear [10, 19–21]. Because 3′ UTRs integrate combinatorial inputs from RBPs and miRs, competition for access to shared regulatory elements has the potential to dynamically reshape post-transcriptional gene expression programs. Despite extensive evidence linking IGF2BP3 to oncogenic gene regulation, whether and how IGF2BP3 directly remodels RISC occupancy on endogenous transcripts *in vivo* remains unresolved. Despite extensive evidence linking IGF2BP3 to oncogenic gene regulation, whether and how IGF2BP3 directly remodels RISC occupancy on endogenous transcripts *in vivo* remains unresolved. To address this question, we mapped AGO2-associated miRNA–mRNA interactions in the presence and absence of IGF2BP3 using AGO2 miR–eCLIP and integrated these data with IGF2BP3 occupancy profiles and functional analyses. These studies reveal widespread changes in AGO2 targeting following IGF2BP3 depletion and provide mechanistic insight into how IGF2BP3 shapes post-transcriptional gene regulation in leukemia.

## Materials and methods

### Cell culture

The human B-ALL cell line SEM (DMZ-ACC 546) was cultured as previously described [22]. All cell lines were maintained in standard conditions in an incubator at 37°C and 5% CO_2_ and cultured in Iscove’s modified Dulbecco’s medium (IMDM) with 10% Fetal Bovine Serum (FBS).

### CRISPR–Cas9-mediated deletion of IGF2BP3 in SEM cells

An IGF2BP3 deletion (I3KO) in SEM cells was introduced using the lentiviral delivery of CRISPR–Cas9 components in a two-vector system and sgRNA sequence as previously described [13, 22].

### Total RNA extraction

SEM cells were collected by centrifugation at 200 × *g* for 5 min and resuspended in TRIzol (Invitrogen). Samples were stored at −80°C until use. Total RNA was extracted using the TRIzol protocol (Invitrogen) for isolation of RNA from cells in suspension. After the total RNA was solubilized in ddH_2_O, one overnight ethanol precipitation step was included for further purification of the total RNA.

### eCLIP sequencing of IGF2BP3 and AGO2

IGF2BP3 eCLIP and AGO2 miR–eCLIP studies were performed by Eclipse Bioinnovations Inc (https://eclipsebio.com/) according to the published enhanced crosslinking immunoprecipitation (eCLIP) and miR–eCLIP protocols [23, 24]. SEM cells were grown and UV crosslinked at 400 mJoules/cm^2^ with 254 nm radiation, flash frozen, and stored until use at −80°C. Crosslinked cell pellets were further processed by Eclipse Bioinnovations for eCLIP using a validated rabbit anti-IGF2BP3 antibody (MBL, RN009P) and Eclipse AGO2 antibody (Santa Cruz Biotech sc-53521). For IGF2BP3, three replicates using 20 million SEM control (NT) cells per replicate and one HEPG2 control were processed, yielding a total of eight libraries (four IP libraries and four size-matched input libraries). For the AGO2 miR–eCLIP dataset, six IP replicate libraries were generated using 17 million cells per replicate: three from SEM NT and three from SEM I3KO. Six size matched inputs (SMI) were generated. Sequencing was performed as Single End 75 base pair reads on the Illumina NextSeq platform.

### eCLIP read processing

Reads from IGF2BP3 and AGO2 eCLIP were pre-processed for quality and adapter trimming (cutadapt; RRID:SCR_011841) followed by alignment to the reference human genome (GRCh38, GENCODE v41 annotation) using STAR v2.7.7a. Repetitive elements were removed by a reverse intersection of all peak files with the repeat masker bed file (downloaded from the University of California Santa Cruz [UCSC] table viewer; RRID:SCR_005780) while PCR duplicate reads were removed, and the aligned files were further processed and analyzed for peaks enriched over the background using Skipper v1.0.087 (RRID:SCR_026260). IGF2BP3 and AGO eCLIP fine-mapped peak sets were filtered for peaks with log2(fold-change) ≥1.0 and ≥3.0, respectively, in terms of mean read counts in IP versus size-matched input. Peaks were annotated using transcript information from GENCODE v41 with the following priority hierarchy to define the annotation of overlapping features: protein coding transcript (CDS, UTRs, and intron), followed by non-coding transcripts (exon and intron).

Differential binding in AGO2 I3KO and NT samples were analyzed using DESeq2 -1.32.0. (RRID:SCR_015687). Count files for each of the replicates were subjected to differential binding to identify statistically significant differences between I3KO and NT. The *P*-values from the Wald tests are displayed as the y-axis of volcano plots, with the log2 fold-change of normalized peak intensities (I3KO over NT) as the *x*-axis.

Bedtools intersect module was used to find the peaks exclusive to NT and I3KO along with the overlapping peaks. Furthermore, the peak counts were normalized to CPM (counts per million). As a background control, uniformly distributed crosslink sites were simulated by generating pseudo-random intervals across the hg38 genome using bedtools. This simulated background models nonspecific crosslinking expected by chance. For each position, observed peak density was scaled relative to the mean simulated background density and centered by subtracting the expected background value, yielding enrichment above the simulated background. These normalized peaks were used for visualization of peak intensities across CDSs and UTRs for AGO2 in NT and KO replicates. Python 3 was used for visualization of metagene plots while the ggplots package from R was used to plot gene enrichment.

### Chimeric AGO2 miR–eCLIP read processing

After sequencing, samples were processed with Eclipsebio’s proprietary analysis pipeline (v1). UMIs were pruned from read sequences using umi_tools (v1.1.1), followed by trimming of 3′ adapters from reads using cutadapt. Reads mapped to a custom database of repetitive elements and rRNA sequences were removed and remaining non-repeat mapped reads were mapped to the hg38 genome using STAR (v2.7.7a). PCR duplicates were removed using umi_tools (v1.1.1). AGO2 eCLIP peaks were identified within eCLIP samples using the peak caller CLIPper (v2.0.1). For each peak, IP versus input fold enrichments and p-values were calculated and mapped to repetitive elements or the genome using bowtie (v1.2.3). The miRNA portion of each read was then trimmed, and the remainder of the read was mapped to the genome using STAR (v2.7.7a). PCR duplicates were resolved using umi_tools (v1.1.1), and miRNA target clusters were identified using CLIPper (v2.0.1). Each cluster was annotated with the names of miRNAs associated with that target. Peaks were annotated using transcript information from GENCODE v41 to define the final annotation of overlapping features: protein coding transcript (CDS, UTRs, and intron), followed by noncoding transcripts (exon and intron). Further, the top differentially expressed miRNAs were selected from small RNA sequencing data and their chimeric read proportions were calculated in NT and I3KO and plotted using GraphPad Prism. To limit our analysis to biologically relevant AGO2 peaks, chimeric AGO2 peaks were overlapped with the total AGO2 peaks from the eCLIP dataset. AGO2 peaks present in both datasets were further used for downstream analysis.

### Quantification of differential chimeric read occupancy

miRNAs exhibiting differences in chimeric read proportions between NT and I3KO were further analyzed to assess the chimeric read occupancy at the mRNA target level for each miRNA. Chimeric peak counts corresponding to the annotated 3′ UTR targets for each miRNA were considered individually for NT and I3KO replicates to calculate the differential chimeric read occupancy (DCRO). DEseq2 was used to identify the significant DCRO across mRNA targets of each miRNA. A matrix of miRNA targets and their corresponding chimeric read occupancy in terms of log2 fold-changes was generated and visualized as a circular heatmap using *ComplexHeatmap* and *circlize* packages in R.

### HOMER motif analysis

The peak bed files from AGO2 miR–eCLIP and IGF2BP3 eCLIP were utilized to identify known and *de novo* motif sequence enrichment for NT and I3KO findMotifsGenome.pl from HOMER2 with default and modified parameters for RNA motifs with a threshold length of 10. To assess motif enrichment across distinct transcript features, peak files were intersected with the reference gene annotation to stratify AGO2 chimeric and IGF2BP3 peaks into CDS, 5′ UTR, and 3′ UTR subsets. Each feature-specific peak set was then analyzed independently with HOMER2 to identify enriched known motifs and discover *de novo* sequence motifs in NT and I3KO conditions. To compare AGO2-associated sequence motifs between I3KO and NT conditions, motifs identified by HOMER2 were converted into MEME-formatted position-weight matrices. Motifs derived from AGO2 chimeric peaks in NT and I3KO cells were processed separately to generate two condition-specific MEME motif libraries. Pairwise motif similarity analysis was then performed using the TomTom module from the MEME Suite [25], which aligns PWMs and computes statistical similarity scores based on Euclidean distance and Pearson correlation metrics. TOMTOM was run with default parameters, reporting the best-matching motifs between NT and I3KO libraries along with associated *P*-values and *q*-values.

### Motif sequence similarity analysis

To quantify the similarity between IGF2BP3 binding preferences and miRNA seed sequences, we used FIMO (Find Individual Motif Occurrences) from the MEME Suite [26] and computed IGF2BP33 motif scores for each canonical miRNA seed family. The IGF2BP3 PWM, derived from *de novo* motif discovery on IGF2BP3 eCLIP peaks, was supplied to FIMO as the query motif. Canonical 7–8 nt seed sequences for all expressed miRNAs were converted into FASTA format and scanned using FIMO with default log-likelihood scoring parameters. For each miRNA seed, FIMO returned the best-scoring I3 motif match along with its associated *P*-value. To integrate sequence-based motif similarity with experimental binding evidence, we extracted IGF2BP3 eCLIP peak intensities for all transcripts targeted by each miRNA family. These values were aggregated to generate an IGF2BP3-binding score per miRNA family. The FIMO-derived motif scores and the eCLIP-derived binding scores were combined into a matrix and visualized as a heatmap, enabling direct comparison of predicted IGF2BP3 motif affinity with experimentally observed IGF2BP3 occupancy across miRNA-regulated transcripts.

### Small RNA (sRNA) sequencing

Total RNA from SEM NT and I3KO cells were used for sRNA library preparation. One microgram of total RNA for each sample was used for sRNA library preparation using the NEXTFLEX small RNA-Seq Kit v3 following the manufacturer’s protocol (Perkin Elmer Applied Genomics). Pooled sRNA sequencing libraries were sequenced on an Illumina HiSeq 4000 at the UC Davis Sequencing Core Facility, generating 100 bp single-end reads.

### mRNA sequencing

Total RNA from SEM NT and I3KO cells was used for mRNA library preparation. Two micrograms of total RNA for each sample was used for mRNA library preparation using the NEXTFLEX Rapid Directional RNA-Seq Kit 2.0 following the manufacturer’s protocol (Perkin Elmer Applied Genomics). Before library preparation, total RNA samples were subjected to Poly(A) selection and purification using the NEXTFLEX Poly(A) Beads Kit 2.0 following the manufacturer’s protocol (Perkin Elmer Applied Genomics). Pooled mRNA sequencing libraries were sequenced on an Illumina NovaSeq S4 at the UC Davis Sequencing Core Facility, generating 150 bp paired-end reads.

### Proteomics analysis

Fifty micrograms of protein lysates were reduced, alkylated, and digested with trypsin using protein aggregation capture (PAC) as described in Batth *et al*. [27]. Digested peptide samples were analyzed by LC-MS/MS on a Vanquish Neo UHPLC system coupled to a Thermofisher Orbitrap Astral mass spectrometer. Data was acquired using a data-independent analysis (DIA) acquisition strategy as described [28]. Data analysis was performed using the DIANN algorithm for peptide and protein identification and quantitation followed by Fragpipe analyst for comparison testing [29, 30].

### Cumulative distribution function

To evaluate whether IGF2BP3 targeted genes exhibit distinct protein-level changes relative to non-targets, we performed a cumulative distribution function (CDF) analysis of RNA and protein log2 fold-change values in I3KO and control SEM cells. Genes were classified as Target or Non-target based on I3 eCLIP peak assignment. For each group, empirical cumulative distribution curves were generated from log2FC values derived from differential RNA and protein expression analysis respectively. Statistical significance between the two distributions was assessed using a two-sample Kolmogorov–Smirnov (KS) test, which quantifies differences in both the location and shape of the distributions. The resulting KS statistic and *P*-value were reported to determine whether I3 bound targets exhibit a systematic shift in RNA or protein abundance relative to nontargets. The plots were visualized using the ggplots package from R.

### Cell cycle analysis using DAPI

SEM cells were washed with and resuspended in PBS. Cells were subsequently stained with a PBS solution containing 4′,6-diamidino-2-phenylindole (DAPI) at a concentration of 1 mg/ml and 0.1% Triton X-100 for 30 min at room temperature. Flow cytometry of DAPI-stained cells was performed on a BD LSRII and analyzed using FlowJo software.

### Generation of miR-181a overexpression cell lines

For the generation of SEM miR-181a overexpression cell lines, the full sequence of miR-18a (5′-AACAUUCAACGCUGUCGGUGAGU-3′) was cloned into the modified retroviral pMSCV vector MGP (Addgene plasmid # 26526; http://n2t.net/addgene:26526; RRID:Addgene_26526). The retroviruses were generated using pGAG/POL helper plasmid and pseudotyped with pVSVG. IGF2BP3 control cells were then stably transduced with retroviral supernatant overexpressing miR-181a and selected with puromycin.

### Quantification of miR-181a by qPCR

miR-181a and RNU66 levels were quantified in SEM cell lines using the TaqMan^™^ MicroRNA Assay (Applied Biosystems) according to the manufacturer’s instructions (assay ID 000480 and 001002, respectively).

### Quantification of cell growth using CellTiter-Glo

Empty MGP vector NT control or miR-181a overexpressing NT control cells were seeded at 1500 cells/well in 96-well plates and cultured for 72 h at 5% CO_2_ and 37°C. Cell growth was quantified using the CellTiter-Glo (Promega) assay according to the manufacturer’s instructions. Luminescence was measured using a Varioskan LUX multimode microplate reader (ThermoFisher). Five technical replicates were prepared for each sample.

### Preparation of recombinant human IGF2BP3 and AGO2 loaded with synthetic miRNA

Recombinant IGF2BP3 tagged with GST was created using the Baculovirus protein expression system. AGO2 loading with synthetic miRNA was combined with affinity purification and performed according to a previously published protocol with modifications [31]. Detailed protein purification methods can be found in the Supplemental Methods.

### *In vitro* transcription and radioactive labeling of RNA substrates for electrophoretic mobility shift assay

The DNA sequence for the EMSA target RNA substrate (Table 1) was cloned and expressed using the pBluescript I KS (+) vector. *In vitro* transcription was performed in the presence of radioactive [α-32] UTP using the MEGAscript T7 High Yield Transcription Kit (Invitrogen) according to the manufacturer’s instructions. After precipitation, the transcribed RNA was run on a 5% polyacrylamide gel, extracted from the gel, and purified. Radioactive labeling efficiency was determined using a Beckman Coulter LS 6500 Multi-Purpose Scintillation Counter.

**Table 1.** List of oligonucleotides used in this study

Oligo name	Sequence	Reference
hsa-miR-let-7a	/5′Phos/UGAGGUAGUAGGUUGUAUAGUU-3′	miRBase
Let-7a RNA capture (2-OMe-RNA modification for each base)	/5′Biotin/mUmCmUmCmGmUmGmCmUmAmCmAmAm UmAmCmGmCmUmAmCmCmUmCmAmAmCmUmCmU-3′	This study
Let-7a DNA competitor	/5′Biotin/AGAGTTGAGGTAGCGTATTGTAGCACGAGA-3′	This study
HMGA2 partial 3′ UTR (EMSA)	5′-TAGCAAAAGGGGGGGGCAATCTCTCTCTGTGTCTTTC TCTCTCTCTCTTCCTCTCCCTCTCTCTTTTCATTGTGTATC AGTTTCCATGAAAGACCTGAATACCACTTACCTCAAATTAAG CATATGTGTTACTTCAAGTAATACGTTTTGACATA AGATGGTTGACCAAGGTGCTTTTCTTCGGCTTGAGTT CACCATCTCTTCATTCAAACTGCACTTTTAGCC-3′	Schneider *et al*. 2019
HMGA2 partial 3′ UTR WT (Luciferase reporter assays)	5′-AAAAGGGGGGGGCAATCTCTCTCTGTGTCTTTCT CTCTCTCTCTTCCTCTCCCTCTCTCTTTTCATTGT GTATCAGTTTCCATGAAAGACCTGAATACCACTTACCTCAAATT AAGCATATGTGTTACTTCAAGTAATACGTTTTGACATAAGA TGGTTGACCAAGGTGCTTTTCTTCGGCTTGAGTTCACCATCTC TTCATTCAAACTGCACTTTTAGCCAGAGATGCAAT ATATCCCCACTACTCAATACTACCTCTGAATGTT-3′	Schneider *et al*. 2019
HMGA2 partial 3′ UTR I3 binding site mutant (Luciferase reporter assays)	5-’AAAAGGGGGGTGTGATCTCTCTCTGTGTCTTTCTCT CTCTCTCTTCCTCTCCCTCTCTCTTTGTGTGGTGTTGTGGTTT CCATGAAAGACCTGAATACCACTTACCTCAAATTAAGGTGTTGTGTTACT TGAAGTAATACGTTTTGTGTGTAGATGGTTG ACCAAGGTGCTTTTCTTTGTGTTGAGTTTGCTGTCTCTTTGTT CAAACTGCACTTTTAGCCAGAGATGCAATATATCC CCACTACTCAATACTACCTCTGAATGTT-3′	Schneider *et al*. 2019

### Electrophoretic mobility shift assay 

Electrophoretic mobility shift assay (EMSA) was performed as previously described in CSH Protocols according to the “Simple Interactions” section [32] using a binding reaction buffer containing 30 mM Tris–HCl pH 8.0, 0.1 M KOAc, 2 mM Mg(OAc)_2_, 0.5 mM TCEP, and 0.005% NP-40 [31, 33].

### Luciferase assays 

Partial *HMGA2* 3′ UTR sequences [17] (Table 1) were ordered as DNA fragments (IDT) and cloned into pMIR-REPORT miRNA Expression Reporter Vector System (ThermoFisher). HEK293T cells were transfected with pMIR-REPORT, *HMGA2* WT, or *HMGA2* I3 mutant containing reporter vectors along with pCG empty vector (EV) or I3 expression vector, as well as pRL-TK control reporter vector (Promega). A total of 4 × 10^5^ HEK293T cells were seeded per well in a 24-well plate. Two hundred nanograms of reporter plasmids were transfected with either 700 ng of pCG EV or 200 ng I3 expression vector supplemented with 500 ng EV to keep CMV promoter concentrations constant. Hundred nanograms of pRL-TK was co-transfected in both conditions. Cotransfections were performed using Lipofectamine 2000 at a 1:2 DNA [μg] to Lipofectamine [μL] ratio and incubated for 24 h. Cell lysates were processed using the Promega Dual Luciferase Reporter Assay kit according to manufacturers instructions and luciferase luminescence was measured using a GloMax Explorer Luminometer (Promega). Relative luciferase activities were calculated as a ratio of Firefly and Renilla raw values with three technical replicates per sample and a total of three independent biological replicates.

### Statistical analysis

A two-sided Fisher’s exact test was employed to determine the enrichment of I3 targets among the pool of differentially expressed genes in the RNA-seq and mass spectrometry data. We performed a contingency analysis comparing transcripts classified as differentially expressed versus non-significant in I3KO cells. Transcripts were stratified based on I3 binding, and counts were tabulated across both categories. Statistical significance was evaluated using a two-sided Fisher’s exact test. Effect size was quantified using the Baptista-Pike method to compute odds ratios and 95% confidence intervals. Similar analysis was performed for mass spectrometry data using differentially expressed and non significant proteins in I3KO. An unpaired *T*-test was used for comparing two conditions. For comparisons of multiple conditions, a two-way ANOVA was performed. To assess whether transcripts co-targeted by miR-181 and I3 were enriched for increased AGO2 occupancy, we constructed a 2 × 2 contingency table comparing AGO2-upregulated transcripts between miR-181/I3 co-targets and all other I3-bound transcripts. miR-181/I3 co-targets were defined as the subset of miR-181 targets overlapping IGF2BP3 eCLIP peaks (*n* = 52). AGO2-upregulated transcripts were identified from chimeric-read analysis in I3-deficient cells (*n* = 50). Enrichment was evaluated using a one-tailed Fisher’s exact test (alternative = “greater”), with significance interpreted as evidence that miR-181/I3 co-targets disproportionately gain AGO2 occupancy relative to other I3 targets. Statistical tests for non-high throughput data were performed using the GraphPad Prism software and are described in the figure legends. A *P*-value of ≤0.05 was considered significant. For quantification of qPCR results, RQ values were normalized to the endogenous control using the 2^–∆∆Ct^ method.

## Results

### IGF2BP3 modulates gene expression at multiple levels in MLL–AF4 B-lymphoblastic leukemia cells

**Figure 1.**
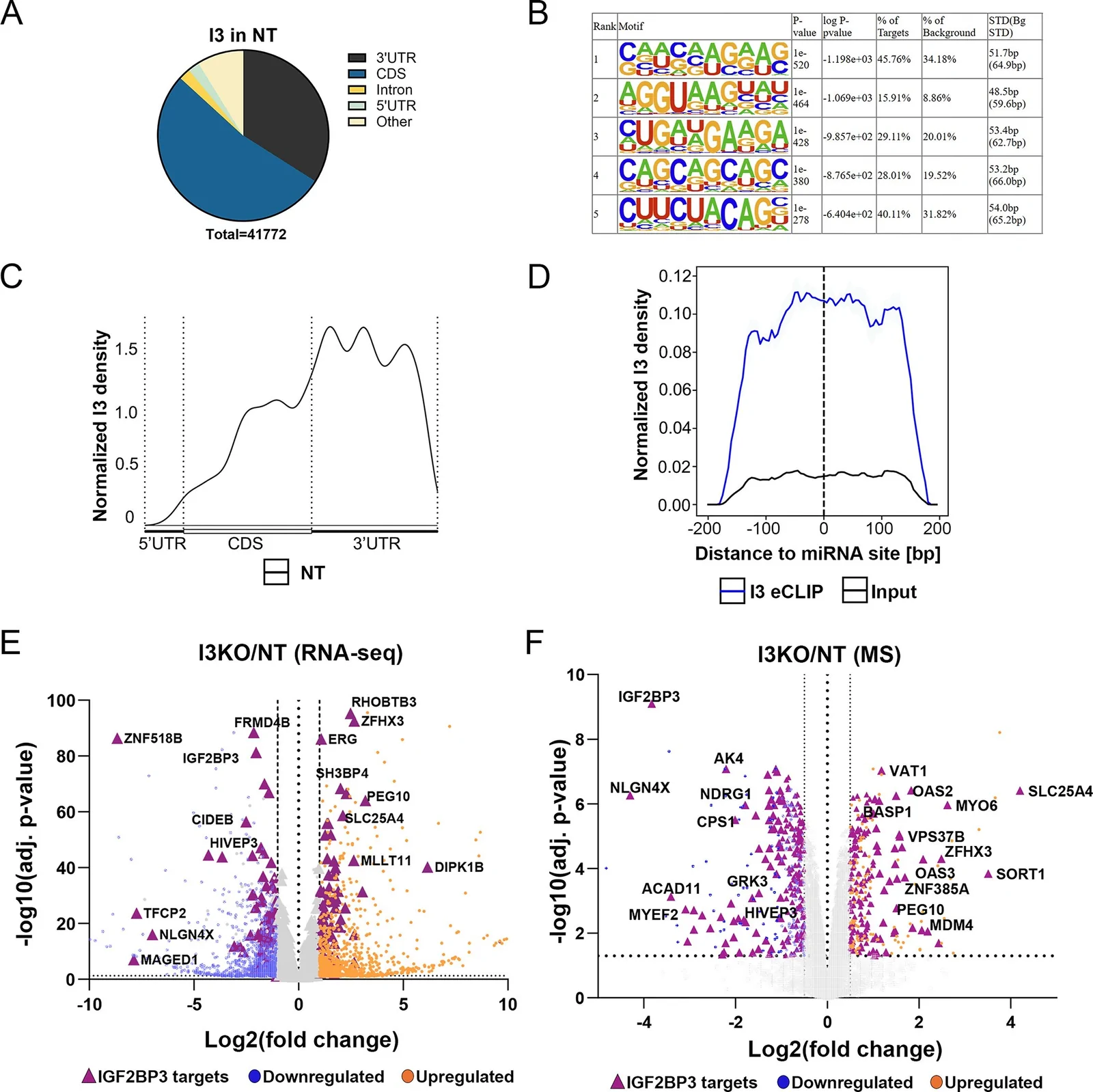
Identification of IGF2BP3-binding sites and differentially expressed genes. (**A**) Distribution of I3 eCLIP peaks across gene features in control (NT) SEM cells. The total number of eCLIP peaks are listed under the pie chart. (**B**) Top five HOMER binding motifs of I3 eCLIP peaks in control SEM cells. (**C**) Metagene plot depicting the relative density of I3 eCLIP peaks in control (NT). Peak counts were normalized as counts per million relative to gene length. (**D**) Metagene plot depicting the normalized density of I3 eCLIP peaks relative to annotated miRNA sites. (**E**) Volcano plot showing differentially expressed transcripts by RNA-seq between control (NT) and I3 knock-out (I3KO) cells, relative to I3KO. Significantly differentially expressed RNAs are depicted as colored dots. Transcripts bound by I3 are marked as triangles. The threshold for differential peaks was defined as < −1 or >1 log2 fold-change, with the *P*-value < 0.05. (**F**) Volcano plot showing differentially expressed proteins by mass spectrometry between control (NT) and I3 knock-out (I3KO) cells, relative to I3KO. Significantly differentially expressed proteins are depicted as colored dots. Transcripts bound by I3 are marked as triangles. The threshold for differential peaks was defined as < −1 or >1 log2 fold-change, with the *P*-value < 0.05.

Previous studies from our group and others have shown that IGF2BP3 (I3) regulates gene expression at various levels [20, 21, 34, 35]. To investigate the impact of I3 in MLL–AF4 translocated B-ALL cells, we established a CRISPR-edited SEM cell line harboring a deletion of I3 (I3KO) and performed eCLIP of I3 as well as RNA-sequencing (RNA-seq) in control and I3KO cells (Fig. 1A, and Supplementary Fig. S1A and B). We performed peak calling analysis for I3 eCLIP in the control using Skipper [36], which identified 41 772 I3 peaks, followed by annotation of I3 peaks across genomic features. Although the majority of peaks were found in the CDS (Fig. 1A), we observe the highest I3-binding density in the 3′ UTR (Fig. 1C). To determine if the previously reported relationship between I3 and RISC [10, 20] is also observed in SEM cells, we calculated I3-binding site density relative to miR target sites. (Fig. 1D). Compared with input, I3 showed a sharp increase in normalized peak density within ±50 nt of the miRNA site, with the strongest signal precisely at position 0. This enrichment indicates that I3 preferentially associates with regions harboring miRNA target sites, consistent with a model in which I3 engages transcripts at or near RISC-accessible elements. The input control remained flat across the same window, confirming that the observed enrichment reflects specific I3 binding rather than background coverage. These positional analyses demonstrate that I3 binds directly over miRNA target sites, placing it in an ideal position to modulate RISC activity on its targets. Having established this physical overlap, we next asked whether I3′s positioning translates into measurable effects on gene output. HOMER [37] revealed several enriched RNA sequence motifs (Fig. 1B), including the top-ranked CA-rich sequence, consistent with previously observed I3-binding preference [17]. Stratifying the motifs by gene feature reveals distinct sequence preferences across 3′ UTR, CDS, and 5′ UTR regions (Supplementary Fig. S1C). Even separated by gene features, I3 eCLIP peaks showed a consistent enrichment for CA-rich motifs in all three genomic features, with the strongest and most specific motif emerging in the 3′ UTR.

To determine how I3 impacts global gene expression, we investigated steady-state mRNA and protein levels in control and I3KO cells (Fig. 1E and F). We discovered a total of 2227 differentially expressed mRNAs (circles), of which just 256 (11.49%) are crosslinked to I3 (purple triangles) in SEM cells (Fig. 1E). By contrast, liquid chromatography-tandem mass spectrometry (LC-MS/MS) (Fig. 1F) identified 741 differentially expressed proteins (circles) of which 391 (53%) are encoded by mRNAs that crosslink to I3 (purple triangles). Overall, the I3 targets were positively correlated at RNA and protein levels (Supplementary Fig. S1D).

Although I3 targets were significantly enriched among both differentially expressed transcripts and proteins (Fisher’s exact test, *P* < 0.0001 for both datasets), the observation that >50% of differentially expressed proteins are encoded by putative I3 target mRNAs suggests that I3-dependent post-transcriptional regulation contributes substantially to gene expression changes in leukemic cells. To further examine the relationship between I3 binding and downstream changes in gene expression, we performed CDF analyses of RNA and protein log2 fold-change values in I3KO and control SEM cells (Supplementary Fig. S1E and F). These analyses revealed both positive and negative shifts among I3 targets relative to nontargets at the RNA and protein levels, consistent with transcript-specific regulatory effects of I3. Together, these findings indicate that I3 influences gene expression beyond steady-state RNA abundance and support a context-dependent role for I3 in post-transcriptional regulation.

### IGF2BP3 reshapes AGO2-binding landscapes on shared target mRNAs

**Figure 2.**
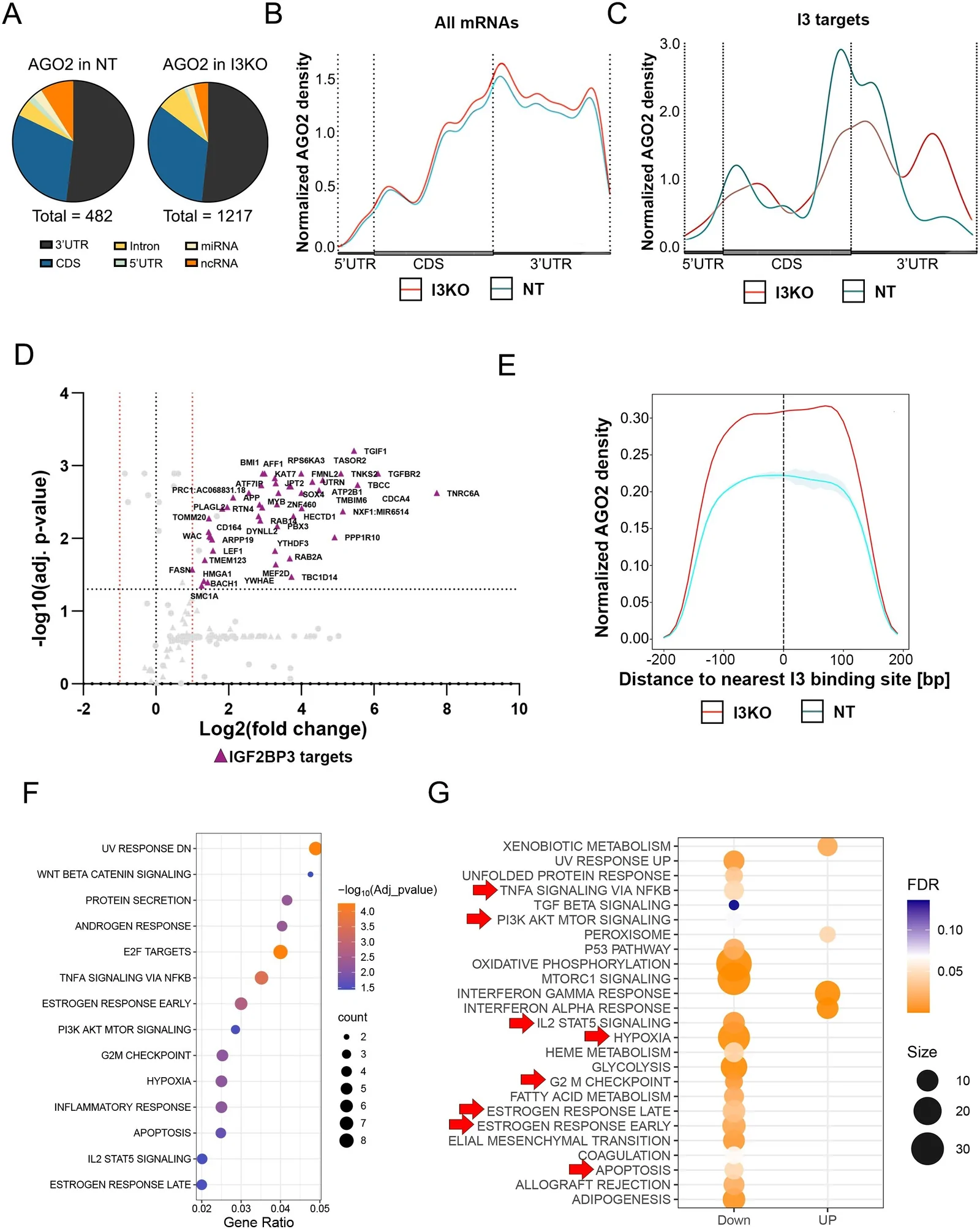
AGO2 binding on oncogenic 3′ UTRs is attenuated by I3. (**A**) Distribution of AGO2 eCLIP peaks across gene features in control (NT) and I3 knock-out (I3KO) SEM cells. Total number of peaks are listed for each condition under the respective pie charts. (**B**) Metagene plot depicting the relative density of AGO2 eCLIP peaks across gene features of all mRNAs (*n* = 928) in control (NT, blue) and I3 knock-out (I3KO, red) SEM cells. Peak counts were normalized as counts per million relative to gene length. (**C**) Metagene plot depicting the relative density of AGO2 eCLIP peaks across gene features of I3 target mRNAs (*n* = 457) in control (NT, blue) and I3 knock-out (I3KO, red) SEM cells. Peak counts were normalized as counts per million relative to gene length. (**D**) Volcano plot showing differential AGO2 eCLIP peaks between control (NT) and I3 knock-out (I3KO) cells, relative to I3KO. AGO2 eCLIP peaks in 3′ UTRs also bound by I3 in NT are marked as triangles (*n* = 47). The threshold for differential peaks was defined as < −1 or >1 log2 fold-change, with the *P*-value < 0.05. (**E**) Metagene plot depicting the relative density of AGO2 eCLIP peaks relative to I3-binding sites in control (NT) and I3 knock-out (I3KO) SEM cells. (**F**) Hallmark enrichment analysis of mRNAs with increased 3′ UTR AGO2 eCLIP peaks in IGF2BP3 knock-out cells (purple triangles from panel D). Significantly enriched pathways had an FDR of <0.05. (**G**) Hallmark enrichment analysis of differentially expressed proteins from our mass spectrometry data. Pathways common with panel (F) are highlighted in red. Significantly enriched pathways had an FDR of <0.05.

To test whether IGF2BP3 constrains RISC engagement on shared 3′ UTR regulatory elements, we performed AGO2 miR–eCLIP in control and I3KO SEM cells. After confirming that loss of I3 does not alter AGO2 protein abundance (Supplementary Fig. S2A, bottom right), we mapped global AGO2–RNA binding sites using miR–eCLIP [24, 38]. Because miR–eCLIP captures all AGO2–RNA associations, including miRNA-independent interactions, we refined the dataset by filtering for AGO2 peaks that contained miRNA–mRNA “chimeric” fusion reads. These chimeric reads represent *direct* miRNA–mRNA targeting events, whereas the full AGO2 peak set also contains indirect or spurious binding events. By applying this filter, we identified 482 and 1217 AGO2 peaks with chimeric reads in control and I3KO cells, respectively, and these chimeric peaks were strongly enriched in 3′ UTRs (Fig. 2A). As expected, the AGO2-binding site density is highest near the stop codon and throughout the 3′ UTR compared to the 5′ UTR or CDS, with little differences observed between conditions (Fig. 2B). By contrast, if we subset the data for transcripts with I3 crosslinking sites, striking differences in AGO2-binding site density between the control and I3KO cell lines emerge (Fig. 2C). In control cells, transcripts marked by I3 crosslinking exhibit elevated AGO2-binding upstream of the stop codon, whereas in I3KO cells there is a significant shift in AGO2-binding sites toward the 3′ UTR. Together these data suggest that I3 protects 3′ UTRs from RISC association.

To determine if there is a direct relationship between I3 and AGO2–RNA interactions we quantified changes in AGO2 occupancy on 3′ UTRs that were also crosslinked to I3 in control cells (Fig. 2D). The volcano plot in Fig. 2D shows differential peak changes in AGO2 eCLIP on I3-target 3′ UTRs (purple triangles) and non-I3 targets (circles). The majority of I3 target transcripts exhibit a significant increase in AGO2 binding in I3 knock-out cells. Notably, 47 out of the 111 I3-bound 3′ UTRs show increased AGO2 binding in the I3KO, while none show significantly decreased AGO2 binding. Because I3 frequently crosslinks to AGO2-bound 3′ UTRs we examined AGO2 occupancy around I3-binding sites to obtain a direct readout of how I3 influences RISC engagement at shared 3′ UTR regulatory elements. If I3 restricts AGO2 access, stabilizes transcripts, or competes with miRNA machinery, these effects should manifest as changes in AGO2 peak density precisely at I3-occupied regions. Metagene profiling of AGO2 chimeric peaks centered on I3-binding sites revealed a distinct positional enrichment of AGO2 signal in both NT and I3KO cells (Fig. 2E). In NT cells, AGO2 peaks showed a modest but reproducible increase within ±50 nt of I3 sites, indicating that AGO2 frequently engages regions bound by I3. Notably, I3KO cells exhibited a substantially higher AGO2 peak density across the entire ±200-nt window, with the strongest enrichment centered directly at the I3-binding site (position 0). This elevated signal in the knockout background suggests increased AGO2 accessibility or loading at I3–proximal regions when I3 is absent. We observe a similar result for our unfiltered AGO2 eCLIP dataset (Supplementary Fig. S2B–H) highlighting the robustness of the effect and demonstrating that I3 antagonizes AGO2 binding on shared 3′ UTR elements and suggests that I3 normally constrains RISC engagement at these sites.

To assess whether changes in AGO2 occupancy upon I3 depletion could be explained by altered sequence preferences or miRNA abundance, we analyzed consensus motifs associated with AGO2-binding sites in control and I3KO cells. Analysis of sequence enrichment surrounding AGO2 peaks by HOMER revealed that many AGO2-associated motifs were shared between control and I3KO cells (Supplementary Fig. S3A), suggesting that broad differences in miRNA expression or RISC loading are unlikely to explain the observed occupancy changes. However, stratified motif analyses across transcript regions (Supplementary Fig. S3B–D) revealed distinct sequence preferences within 3′ UTRs following I3 depletion. In I3KO cells, AGO2 occupancy shifted toward 3′ UTR regions enriched for sequence motifs that closely resembled the I3 3′ UTR motif (Supplementary Fig. S3E), whereas AGO2-bound regions in NT cells exhibited more heterogeneous sequence features, including canonical miRNA seed-like elements. These findings indicate that the sequence environments surrounding AGO2 occupancy undergo selective remodeling following I3 depletion. In particular, AGO2-associated 3′ UTR motifs in I3KO cells closely resemble motifs enriched within I3 eCLIP peaks, suggesting that loss of I3 permits RISC engagement at regulatory elements normally occupied by I3. Importantly, small RNA-seq analysis showed no significant changes in the abundance of miRNAs detected in our miR–eCLIP dataset (Supplementary Fig. S4A), further supporting the conclusion that altered AGO2 motif architecture in I3KO cells is not driven by changes in miRNA expression.

Given the widespread changes in AGO2 occupancy observed following I3 loss, we next asked whether these effects could be explained by alterations in transcript architecture rather than changes in RISC targeting. To address this we additionally analyzed alternative polyadenylation and 3′ UTR isoform usage between control and I3KO cells (Supplementary Methods). We observed minimal global changes in 3′ UTR organization, with only a small subset of I3 targets showing altered isoform usage (Supplementary Fig. S4B). These findings support the conclusion that the observed AGO2 occupancy changes primarily reflect altered RISC targeting rather than widespread transcript remodeling.

Having established that altered AGO2 binding is not driven by major changes in transcript architecture, we next examined whether these changes are associated with downstream effects on protein expression. Specifically, we assessed protein-level changes among AGO2-bound transcripts linked to the pro-proliferative pathways identified above. Hallmark pathway enrichment analysis of differentially expressed proteins from our mass spectrometry dataset (Fig. 2G) revealed significant downregulation of multiple pathway components in I3KO cells, including genes belonging to several pathways enriched in the AGO2 occupancy analysis (Fig. 2F). This further suggests that I3 interferes with AGO2-mediated repression of genes associated with pro-proliferative pathways. Finally, to explore the mechanistic basis of this effect, we investigated whether the increased AGO2–3′ UTR interactions observed in I3KO cells occur at functionally relevant regulatory regions. We, therefore, quantified AGO2 crosslinking density relative to annotated miRNA target sites in control and I3KO cells (Supplementary Fig. S4C). Interestingly, deletion of I3 resulted in a significant increase (*P*-value = 3.5e-2) in AGO2 crosslinking near annotated miRNA target sites relative to control cells.

Features such as local AU-richness are known to promote miRNA targeting. To assess whether I3 loss alters the intrinsic sequence environment of AGO2 motif instances, we performed additional sequence composition analyses (Supplementary Methods) surrounding AGO2-associated motifs and found no major differences in AU-richness between control and I3 knockout conditions. These results indicate that the observed changes in AGO2 occupancy are unlikely to be driven simply by altered sequence composition. (Supplementary Fig. S4D).

To assess the functional relevance of transcripts whose AGO2 binding becomes I3-dependent, we performed pathway enrichment analysis using the Hallmark gene sets (Fig. 2F). I3-regulated AGO2 targets were significantly enriched for pro-proliferative and oncogenic programs, including apoptosis, G2M checkpoint, E2F targets, TNFA signaling via NF-κB, WNT/β-catenin, PI3K–AKT–mTOR, and IL2–STAT5 signaling. Similar enrichment patterns were observed in the unfiltered AGO2 dataset (Supplementary Fig. S2G). Browser snapshots of representative transcripts such as *HOXA7* and *BCL2L11* illustrate the increased AGO2 peak intensity in I3KO cells (Supplementary Fig. S2H). Collectively, these findings support a model in which I3 restricts AGO2 binding at 3′ UTRs of proliferation-associated mRNAs, thereby modulating post-transcriptional programs relevant to leukemic growth.

### IGF2BP3 alters the 3′ UTR occupancy of several tumor suppressive and oncogenic miRNAs

Our data suggest that I3 antagonizes AGO2 interactions at select 3′ UTRs. To determine whether I3 broadly influences all miRNAs or selectively alters the targeting behavior of specific miRNAs, we interrogated miR–eCLIP datasets for I3-dependent changes in chimeric read representation. Total chimeric read counts did not differ significantly between control and I3KO cells (Supplementary Fig. S5A). However, normalization of miRNA-associated chimeric reads revealed significant differences in the representation of miR-181a, miR-20a, miR-17, and miR-19b between the two conditions (Supplementary Fig. S5B). Whereas miR-181a exhibited increased 3′ UTR chimeric reads in I3KO cells, miR-20a, miR-17, and miR-19b showed the opposite trend. Importantly, small RNA sequencing demonstrated that steady-state levels of these miRNAs were not altered following I3 depletion (Supplementary Fig. S5C), indicating that the observed differences reflect changes in target occupancy rather than miRNA abundance.

**Figure 3.**
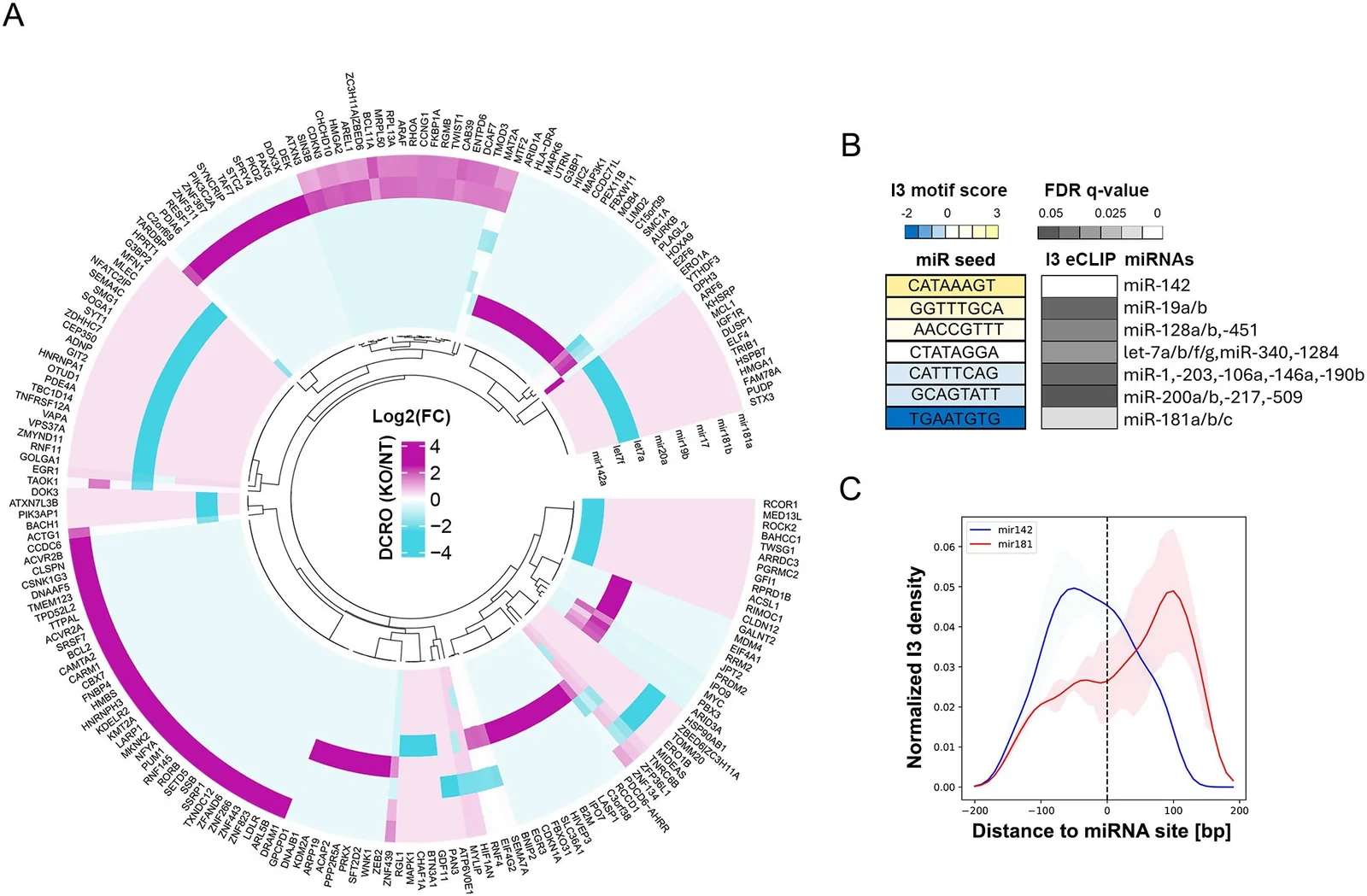
IGF2BP3 remodels the targeting landscape of cancer-related miRNAs. (**A**) Circle plot depicting DCRO of miR-181a/b, miR-17, miR-19b, miR-20, let-7a, let-7f, and miR-142 between control (NT) and I3 knock-out (I3KO) cells, relative to I3KO with a log2FC of +1 and −1 and *P*-value < 0.05. (**B**) I3 Motif-Seed Similarity across miRNA Families. FIMO-based PWM scanning of canonical miRNA seed sequences using the I3 motif. Heatmap shows the highest-scoring I3 motif match for each miRNA seed and corresponding I3 eCLIP binding across miRNA target transcripts. miRNA families with strong I3 motif similarity exhibit higher I3 occupancy on their targets. (**C**) Metagene profile with I3 peaks for miR families exhibit localized I3 enrichment near their target sites, with miR-142 showing a centered I3 enrichment peak around the seed site and miR-181 showing maximal enrichment immediately downstream.

To define transcript-specific changes in miRNA targeting, we quantified DCRO for individual miRNA 3′ UTR interactions (Fig. 3A). Across the selected miRNAs, we identified 200 transcripts exhibiting significant DCRO. miR-181a displayed the greatest number of DCRO events, all of which represented increased AGO2 occupancy in I3KO cells (Fig. 3A and Table 2; Supplementary Fig. S5D). In contrast, let-7 family members, miR-20a, and miR-19b exhibited both increased and decreased DCRO events, whereas miR-142 and miR-17b exclusively showed decreased DCRO in I3KO cells (Fig. 3A and Table 2). These findings indicate that I3 exerts transcript and miRNA-specific effects on RISC engagement rather than uniformly antagonizing miRNA targeting.

**Table 2.** Number of DCRO events for the selected microRNAs relative to IGF2BP3 knock-out cells

microRNA	Number of targets	DCRO Up	DCRO Down	Total DCRO	Total DCRO on I3 targets
miR-181a	131	61	0	61	52
miR-181b	60	39	7	46	37
let-7a	51	10	15	25	24
let-7f	48	23	8	31	28
miR-20a	170	16	5	21	17
miR-19b	39	11	27	38	33
miR-142	402	0	11	11	9
miR-17	162	0	5	5	4

To investigate whether sequence compatibility contributes to the selective effects of I3 on miRNA targeting, we compared I3-binding motifs with canonical miRNA seed sequences. Several miRNAs exhibiting DCRO, including miR-142, miR-19, and let-7 family members, showed strong positive I3 motif scores, whereas miR-181 displayed relatively weak motif similarity to the I3 motif (Fig. 3B). Consistent with these observations, metagene analyses revealed pronounced I3 enrichment over miR-142 target sites, whereas I3 occupancy relative to miR-181 target sites was shifted downstream (Fig. 3C).

To determine whether these patterns were representative of broader miRNA classes, we extended the analysis to additional miRNA families exhibiting DCRO (Supplementary Fig. S5G). Distinct I3-binding architectures emerged across different miRNA target sites. miR-142 exhibited the strongest and most sharply centered I3 enrichment, while miR-17, miR-19b, and let-7 family members displayed broader and lower-amplitude enrichment profiles. In contrast, miR-181 target sites consistently exhibited a downstream-shifted I3 peak. These observations suggest that I3 associates with different classes of miRNA-regulated 3′ UTR elements through distinct spatial arrangements, ranging from direct overlap at some target sites to adjacent positioning at others.

Unexpectedly, despite exhibiting the strongest motif similarity to I3 and the most pronounced I3 enrichment among the miRNA families examined, miR-142-associated transcripts did not overlap with I3-bound targets that gained AGO2 occupancy following I3 loss. In contrast, miR-181-associated transcripts showed robust AGO2 redistribution despite relatively weak motif similarity to I3 (Supplementary Fig. S6C). Together, these findings indicate that sequence compatibility alone is insufficient to predict direct I3-mediated RISC remodeling and support the existence of multiple modes of I3-dependent regulation.

### I3-dependent remodeling of miR-181 targeting influences leukemic growth

**Figure 4.**
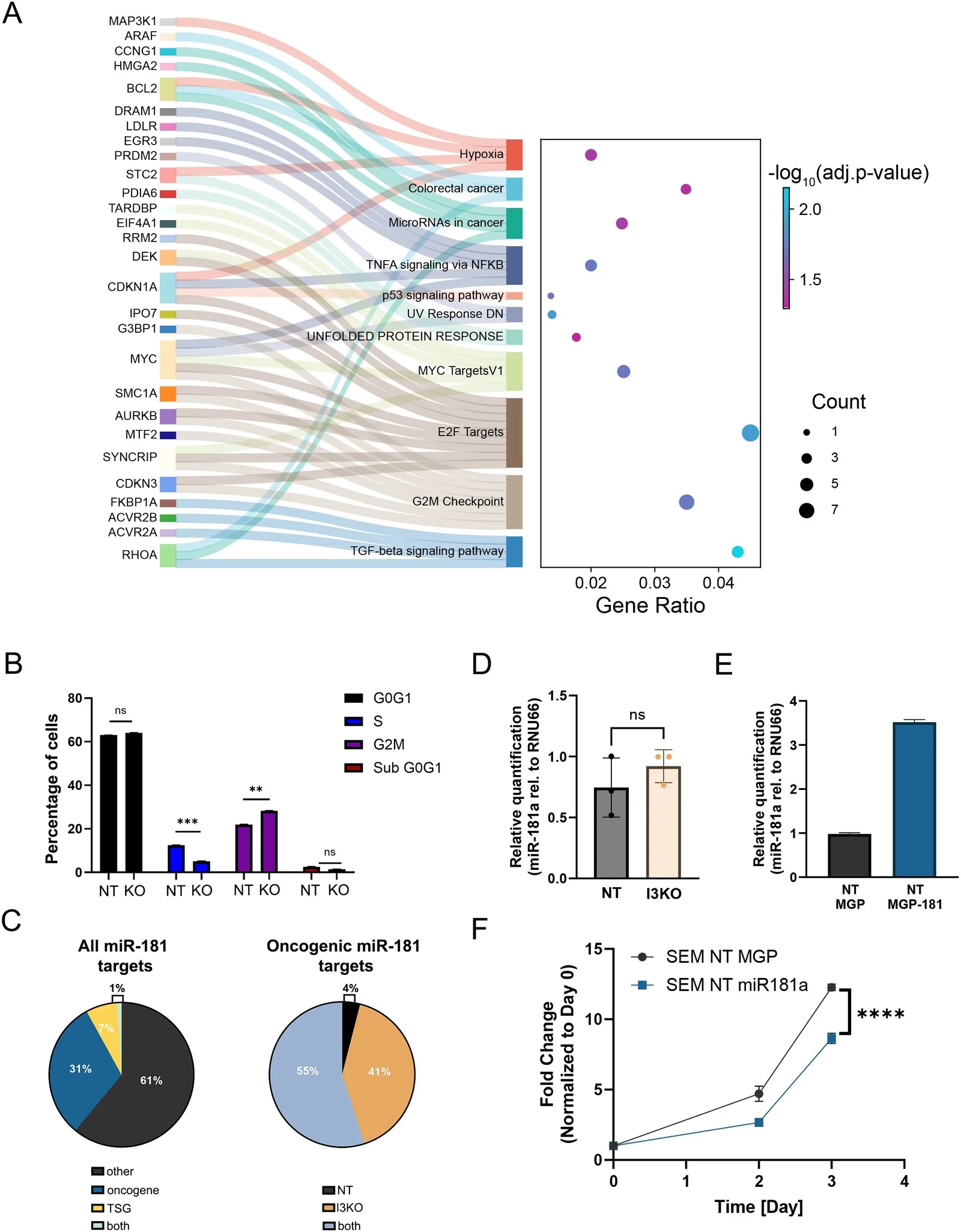
IGF2BP3 remodels the targeting landscape of cancer-related miRNAs. (**A**) Hallmark enrichment analysis of mRNAs with increased DCRO from Fig. 3. Significantly enriched pathways had an FDR of <0.05. Genes part of the enriched pathways are annotated and connected by a Sankey plot. (**B**) Cell cycle analysis on control and I3 knock-out (KO) cells by DAPI staining. The percentage of cells at each stage is shown. (**C**) The proportion of miR-181 targets annotated as either oncogenes or tumor suppressors according to the TSGene database is shown on left. The proportion of oncogenic miR-181 targets from the left pie chart bound by miR-181 in either control (NT, black), I3 knock-out (I3KO, orange), or both (blue) is shown on the right. (**D**) Quantification of miR-181 levels in control (NT) and I3 knock-out (I3KO) SEM cells. RQ values of miR-181 relative to the small RNA control RNU66 are plotted. Paired *t*-test; *P*-value: 0.497. (**E**) Quantification of miR-181a in control cells with empty vector MGP (NT MGP, black), and control with miR-181 overexpression (NT MGP-181a, blue). RQ values normalized to the endogenous small RNA control RNU66 using the 2–ΔΔCt method are plotted for each condition. (**F**) Quantification of cell growth using the CellTiter-Glo assay of SEM cells with empty vector MGP (NT MGP) and control with miR-181 overexpression (NT miR181a). Fold-change of luminescence relative to day 0 is plotted for each condition. Two-way ANOVA; *P*-value < 0.0001.

Hallmark enrichment analysis of all 200 mRNA targets in Fig. 3A revealed that mRNAs with increased DCRO in the I3KO have an enrichment for cancer-related processes, including MYC-related genes (MYC-Targets V1), p53 signaling, and regulation of cell cycle (E2F targets, G2M checkpoint) among others (Fig. 4A). Due to the enrichment of cell cycle pathway terms, we evaluated the impact of I3 expression on cell cycle progression by quantifying the DNA content of DAPI-stained cells using flow-cytometry (Fig. 4B and Supplementary Fig. S5H). In control cells, we observed a significant increase in the percentage of cells in S-phase compared to the I3KO cells. Conversely, we observed a decreased percentage of cells in the G2M phase. This result recapitulates previous studies that also showed an I3-dependent change in cell cycle progression [39, 40]. In contrast to mRNAs with increased DCRO, there were no significantly enriched Hallmark processes for mRNAs with decreased DCRO in the I3KO, but we did find several significantly enriched KEGG pathways, albeit with low gene counts (Supplementary Fig. S5I). To determine if I3 has a direct effect on miRNA occupancy, we quantified the number of DCRO on mRNAs bound by I3 and observed a similar result than for the previous set of mRNAs (compare Supplementary Fig. S6A with Fig. 3A). These transcripts are enriched for GO Biological processes belonging to transcriptional regulation, regulation of cell cycle, and signaling pathways, among others (Supplementary Fig. S6B). We also found that a significant fraction of miRNA targets with increased DCRO also exhibited increased AGO2 chimeric-read occupancy in the I3 knockout background (compare Supplementary Fig. S6C with Fig. 2D). For example, among the 52 transcripts co-targeted by miR-181 and I3 that exhibited increased DCRO, 15 showed increased AGO2 occupancy, representing the strongest enrichment (Fisher exact *P*-value = 0.00231) among all miRNAs analyzed. This was followed by miR-17, let-7f, miR-20a, and let-7a, which contributed 8, 7, 7, and 6 co-targeted transcripts with elevated AGO2 occupancy, respectively. By contrast, no 3′ UTRs with elevated AGO2 occupancy contained chimeric reads for miR-142. The absence of shared I3-miR-142 targets with increased AGO2 occupancy underscores that the AGO2 gains observed in chimeric reads are not driven by miR-142 activity. Further, we also correlated these miRNA targets having increased AGO2 occupancy with their expression at RNA and protein level (Supplementary Fig. S6D and E). The downregulated targets at protein level were enriched in cancer hallmark pathways like PI3K–AKT–mTOR signaling and hypoxia (Fig. 2). Together, these data indicate that the increase in AGO2 occupancy in I3KO cells is predominantly driven by miR-181, with secondary contributions from the miR-17 family, let-7 family, and miR-20a and supports a model in which I3 loss disproportionately enhances AGO2 engagement at miR-181-regulated 3′ UTR elements.

miR-181 is hypothesized to function as a tumor suppressor in several types of cancer [41]. In our dataset, miR-181a exhibits the highest number of DCROs, all of which represent increased occupancy in I3KO cells. To determine whether I3 interferes with miR-181 targeting of oncogenic transcripts, we quantified changes in miR-181 occupancy using our chimeric read dataset. We found that 31% of 3′ UTRs targeted by miR-181 in either I3KO or control cells correspond to oncogenes (Fig. 4C, left panel). Notably, deletion of I3 resulted in a significant increase in miR-181 association with oncogenic transcripts (Fig. 4C, right panel). Previous studies from our group and others demonstrated that loss of I3 consistently impairs leukemic cell growth across multiple leukemia models [10, 11, 13]. The increased miR-181 occupancy observed in I3KO cells suggested that I3 may antagonize miR-181 activity. To test this hypothesis, we generated SEM cell lines overexpressing miR-181a (Fig. 4D and E). Consistent with the increased miR-181 occupancy observed in I3KO cells, miR-181a overexpression significantly reduced the growth of SEM cells relative to controls (Fig. 4F). These findings support a model in which I3 antagonizes miR-181 activity in leukemic cells and suggest that enhanced miR-181-mediated repression contributes to the growth defects associated with I3 depletion.

### IGF2BP3 restricts AGO2 engagement on shared 3′ UTR regulatory elements

**Figure 5.**
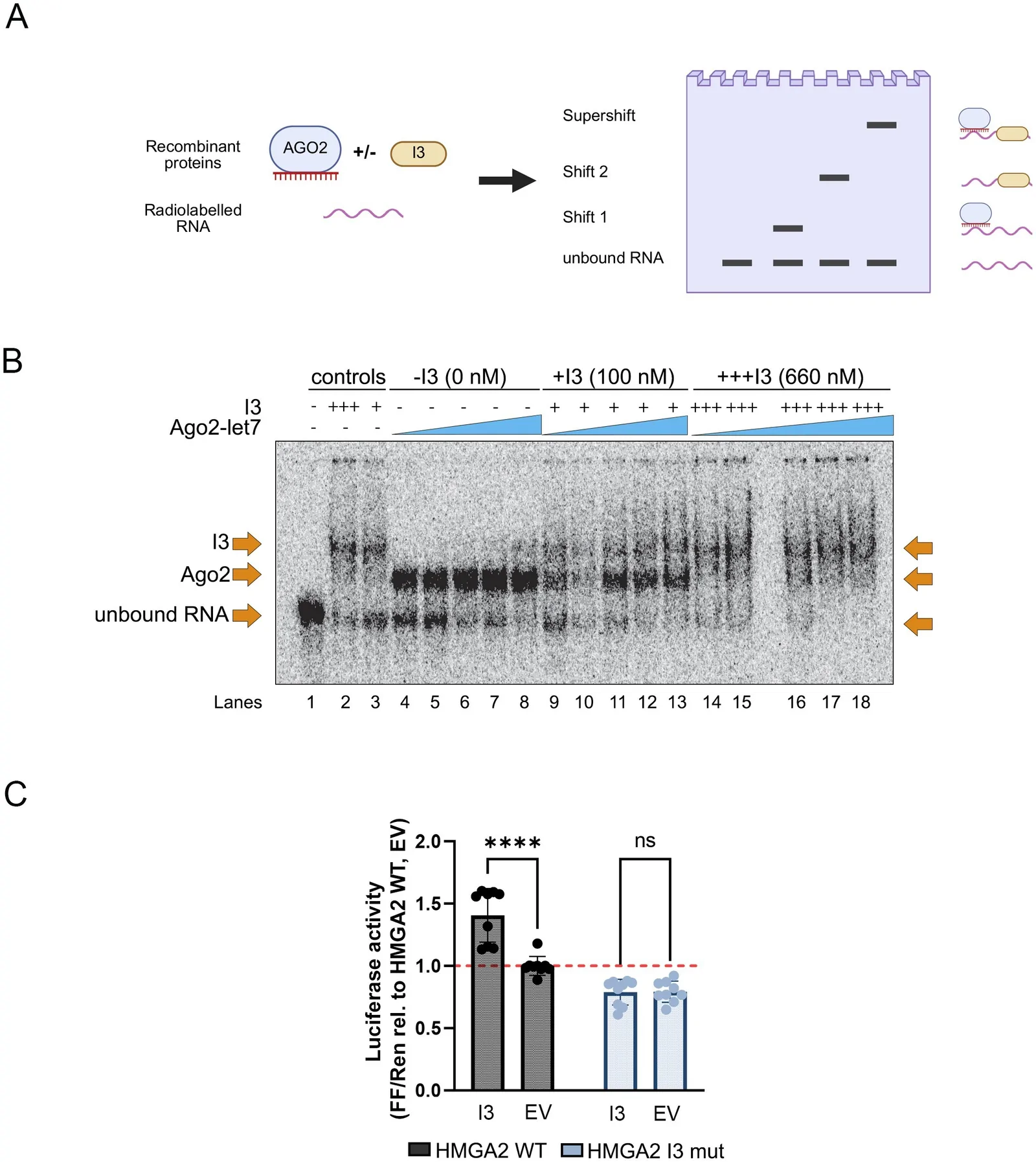
IGF2BP3 directly competes with AGO2 *in vitro*. (**A**) Schematic overview of the EMSA experiment. Recombinant I3 and AGO2 were affinity purified and incubated with radioactively labeled RNA. For AGO2, the affinity purification was combined with loading of a synthetic microRNA. Created in BioRender. Kabbani, L. (2026) https://BioRender.com/4hzrrym. (**B**) EMSA using recombinant I3 and AGO2 loaded with synthetic let-7a microRNA. A partial 3′ UTR sequence from the oncogene HMGA was used as the RNA substrate. Lane 1 = no protein control, Lane 2 = saturating (660 nM) I3 only, Lane 3 = sub-saturating (100 nM) I3 only, Lanes 4–8 = AGO2–let-7a titration without I3, Lanes 9–13 = AGO2–let-7a titration with sub-saturating I3, Lanes 14–18 = AGO2–let-7a titration with saturating I3. (**C**) Luciferase reporter assay using a partial HMGA2 3′ UTR sequence in HEK293T cells. Cells were either transfected with I3 expression plasmid (I3) or empty vector (EV). A substrate carrying mutations in I3-binding sites (I3 mut) was compared to the WT. Luciferase activities were measured as a ratio of Firefly/Renilla activity and compared to cells transfected with the HMGA2 WT and EV expression construct (two-way ANOVA; *P* < 0.0001 and *P* = 0.1546, respectively; ****, *P* < 0.0001; ns, not significant).

Our genomic analyses suggested that I3 restricts AGO2 engagement at shared 3′ UTR regulatory elements. To directly examine the molecular basis of this relationship, we generated recombinant I3 and AGO2-containing RISC complexes for EMSAs (Fig. 5A). Recombinant human I3, as well as human AGO2 loaded with defined miRNAs, were expressed in Sf9 insect cells and affinity purified using epitope tags (Supplementary Fig. S7A and B). Because AGO2 bound to a guide miRNA is sufficient for target recognition, we used these minimal RISC complexes to test whether I3 alters AGO2 association with a shared RNA substrate [42, 43]. RNA substrates were transcribed *in vitro* and radiolabeled at the 5′ end. As a model substrate, we selected a 225-bp fragment from the *HMGA2* 3′ UTR previously shown to bind I3 in EMSA assays [17]. Because this region contains let-7 recognition sites, we generated recombinant AGO2 loaded with let-7 miRNA and performed EMSAs in the presence of increasing concentrations of I3 (Fig. 5B). AGO2–let-7 complexes were titrated in a 2-fold dilution series ranging from 0.025 to 2 nM, with controls lacking either I3 or AGO2 included in parallel. As I3 concentration increased, the intensity of the AGO2-associated shifted band reproducibly decreased across independent experiments (*n* = 3), consistent with competitive binding between I3 and AGO2-containing RISC complexes on the *HMGA2* 3′ UTR. Because recombinant I3 was purified as a GST fusion protein, we additionally tested GST alone as a control and observed no detectable RNA binding (Supplementary Fig. S7C). Importantly, mutation of previously characterized I3 recognition motifs within the *HMGA2* 3′ UTR markedly reduced GST-I3 binding (Supplementary Fig. S7D), supporting the specificity of the interaction. Together, these biochemical data support the idea that direct competition between I3 and AGO2-containing RISC complexes contributes to I3-dependent regulation at select 3′ UTR regulatory elements. To determine whether these I3–RNA interactions also contribute to post-transcriptional regulation in cells, we next performed luciferase reporter assays using wild-type and I3-binding-site mutant *HMGA2* 3′ UTR reporters in HEK293 cells (Fig. 5C). Ectopic I3 expression increased activity of the wild-type *HMGA2* reporter, whereas mutation of the I3 recognition motifs largely abolished this effect. These findings demonstrate that I3-binding sites within the *HMGA2* 3′ UTR are required for I3-dependent regulation of reporter expression *in vivo*.

## Discussion

Previous studies implicated I3 in regulating miRNA-mediated gene silencing, but how this oncofetal RNA-binding protein influences RISC targeting remained unresolved [10, 17, 20]. Here, by combining miR–eCLIP, AGO2 occupancy profiling, and biochemical competition assays, we demonstrate that I3 and RISC converge on overlapping regulatory elements within 3′ UTRs of oncogenic transcripts. Metagene analyses revealed that I3 binding is concentrated at miRNA target regions, whereas loss of I3 resulted in increased AGO2 occupancy and redistribution of AGO2 binding around these sites. Together with our biochemical data, these findings support a model in which I3 acts as a transcript-selective antagonist of RISC, limiting AGO2 access to shared 3′ UTR elements and thereby reshaping the post-transcriptional regulatory landscape of leukemia cells.

Our data further suggest that selective RISC remodeling is influenced by the underlying sequence architecture of I3-bound 3′ UTRs. Motif enrichment analyses revealed that the predominant I3-binding motif closely resembles the sequence environment surrounding AGO2-enriched sites identified in I3KO cells, indicating that transcripts undergoing AGO2 redistribution contain shared GU-rich regulatory elements (Supplementary Fig. S3C and E). In control cells, our data indicate that these regions are preferentially occupied by I3, whereas loss of I3 results in increased AGO2 occupancy at the same sites. These findings suggest that susceptibility to I3-dependent RISC regulation is encoded, at least in part, by local sequence features that favor competitive occupancy of shared 3′ UTR elements. Consistent with this model, transcripts containing these motifs exhibit evidence of direct competition between I3 and AGO2, with occupancy likely determined by the relative affinity of each factor for the local RNA environment (Supplementary Fig. S3E). Notably, despite strong motif similarity between miR-142-associated sites and the I3 motif, transcripts exhibiting altered miR-142 occupancy did not overlap with changes in AGO2 occupancy in I3-bound 3′ UTR targets, suggesting that sequence compatibility alone is insufficient to predict direct I3-mediated RISC remodeling.

Biochemical analyses provide direct support for this model. EMSAs demonstrated that purified I3 competes with AGO2 for binding to shared RNA substrates and displaces AGO2 in a concentration-dependent manner. Consistent with these findings, loss of I3 increased AGO2 occupancy on proliferation-associated transcripts *in vivo*, whereas addition of purified I3 reduced AGO2 binding *in vitro*. Together, these findings support a model in which direct competition contributes to selective RISC remodeling by I3 [21]. The functional consequences of this competition are reflected in the RNA and protein expression changes observed following I3 deletion. CDF analyses revealed both positive and negative shifts in RNA and protein abundance among I3 targets, with a subset of transcripts exhibiting reduced RNA and protein abundance in I3KO cells, consistent with enhanced RISC-mediated repression. These observations support a model in which redistribution of AGO2 following I3 loss contributes directly to transcript-specific post-transcriptional regulatory changes that favor suppression of oncogenic gene expression programs (Fig. 2G). Although changes in RNA and protein abundance are consistent with altered RISC-mediated regulation, the present study does not distinguish between effects on mRNA stability and translational efficiency. Instead, our data support a model in which IGF2BP3 regulates steady-state target expression by modulating RISC accessibility at select 3′ UTR regulatory elements.

While our prior work predicted direct competition between I3 and specific miRNAs for overlapping binding sites [20], the present study reveals a more complex mode of regulation. Consistent with earlier predictions, miR–eCLIP identified I3-dependent interactions involving miR-181a/b, miR-17, miR-19b, and miR-20a on transcripts (Fig. 3A). However, not all changes in miRNA occupancy were consistent with simple antagonism. We observed examples in which I3 both promoted and inhibited binding of specific miRNAs, including miR-142a and let-7 family members, and identified instances of apparent miRNA exchange on shared 3′ UTRs. For example, deletion of I3 favored replacement of miR-17 by let-7 on the *SEMA7A, EIF4G2*, and *RNF4* transcripts (Fig. 3A). These findings suggest that I3 influences miRNA accessibility through mechanisms beyond direct competition. One possibility is that I3 remodels local 3′ UTR RNA structure, thereby altering accessibility of nearby miRNA recognition elements. A similar mechanism has been described for the polypyrimidine tract-binding protein (PTB), which both antagonizes and facilitates miRNA interactions through RNA structural remodeling during neuronal differentiation [44]. Although additional experiments will be required to directly test this model, our data are consistent with a role for I3 as a dynamic regulator of miRNA accessibility that can either restrict or facilitate RISC engagement in a transcript-dependent manner.

The biological consequences of I3-mediated RISC remodeling are particularly evident for miRNAs with established roles in hematopoiesis and leukemia. Although miR-181 has been reported to function as either a tumor suppressor or oncogene depending on cellular context [45–47], our data indicate that I3 antagonizes miR-181 binding to oncogenic transcripts in human leukemic B cells. Consistent with this model, overexpression of miR-181a partially phenocopies the growth defect observed following I3 deletion [10, 11, 13, 20], consistent with the possibility that elevated miR-181 activity can overcome I3-mediated restriction. In contrast, let-7 family members are well-established tumor suppressors, whereas miR-17, miR-19b, and miR-20a belong to the oncogenic miR-17∼92 cluster [48–51]. The distinct effects of I3 on these miRNAs further support a transcript- and miRNA-specific mode of regulation. Notably, miR-181 and miR-142 are among the most abundant miRNAs detected in both our small RNA sequencing and miR–eCLIP datasets (Supplementary Fig. S3A and C), underscoring the physiological relevance of I3–RISC interactions in leukemic cells. Although competition between RNA-binding proteins and RISC has been described for factors such as Musashi1, HuR, and PTB [44, 52–55], our findings identify I3 as a major regulator of this process in leukemia. Together, these data position I3 as a central post-transcriptional regulator of leukemic gene expression programs.

In summary, our work defines a core post-transcriptional mechanism by which I3 shapes leukemic gene expression programs. By regulating access of RISC to shared 3′ UTR regulatory elements, I3 selectively remodels miRNA target interactions and influences the expression of transcripts that support leukemic growth. Rather than acting solely as an mRNA stabilizing factor, our findings support a model in which I3 functions as a transcript-selective regulator of RISC accessibility that reshapes the miRNA regulatory landscape of leukemia cells. These results resolve longstanding questions regarding the relationship between I3 and miRNA-mediated repression and establish a broader framework for understanding how RNA-binding proteins control gene expression through dynamic interactions with the RISC machinery.

## Supplementary Material

zcag025_Supplemental_Files

## Data Availability

mRNA sequencing of NT and I3KO cells, as well as IGF2BP3 eCLIP were previously published [34] and can be accessed under the BioProject accession number PRJNA1191523. Small RNA sequencing and AGO2 eCLIP data generated for this study can be accessed under the BioProject accession number PRJNA1339044. Liquid Chromatography-Tandem Mass Spectrometry data generated for this study can be accessed under the accession number MSV000099159 in the MASSive repository.

## Notes

Present Address: Division of Rheumatology, Rosalind Russell and Ephraim P. Engleman Arthritis Research Center, Department of Medicine, University of California, San Francisco, CA 94117, United States
