# Supplementary material for: IGF2BP3 remodels RISC occupancy to control microRNA targeting in leukemia
Source: NAR Cancer. 2026 Sep 23;8(3):zcag025. doi: 10.1093/narcan/zcag025 (PMC13598244; doi:10.1093/narcan/zcag025)
Supplement: Supplementary file 1 [file zcag025_supplemental_files.zip › Combined_supplementary_figures_final_new.pdf]

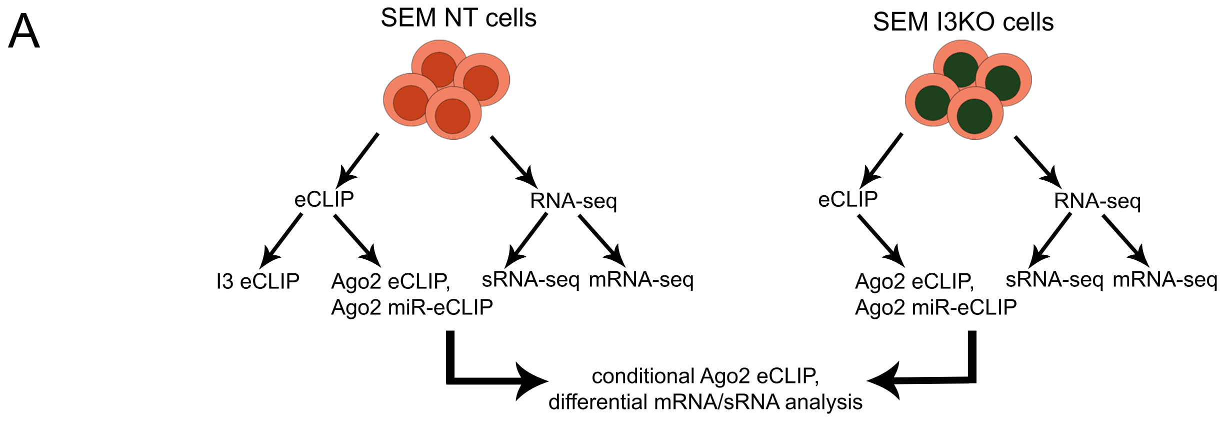

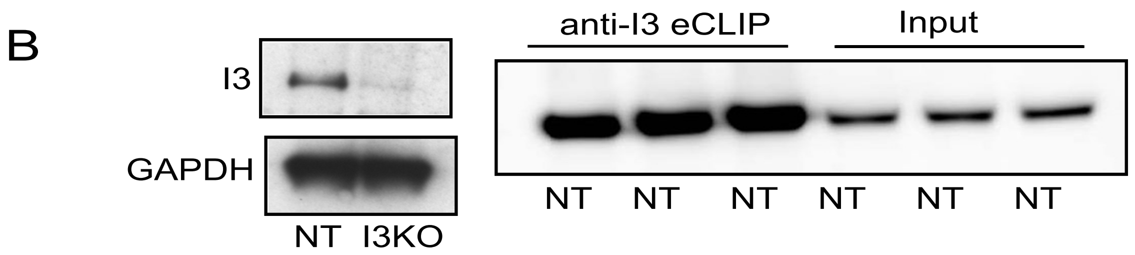

C

I3_3'UTR

Rank	Motif	P-value	log P-pvalue	% of Targets	% of Background	STD(Bg STD)
1	AAAGCUGUAU	1e-75	-1.727e+02	44.45%	36.26%	55.2bp (66.0bp)
2	AACUUUUCAG	1e-67	-1.564e+02	29.60%	22.70%	54.3bp (67.8bp)
3	AUUUGUACUG	1e-61	-1.421e+02	12.79%	8.29%	54.4bp (65.4bp)
4	UUUUAUACAA	1e-56	-1.294e+02	30.66%	24.28%	53.9bp (63.9bp)
5	CUUAACAGUU	1e-55	-1.278e+02	30.89%	24.53%	55.8bp (66.8bp)

I3_CDS

Rank	Motif	P-value	log P-pvalue	% of Targets	% of Background	STD(Bg STD)
1	AAGGUAAGUA	1e-706	-1.627e+03	27.20%	10.94%	38.4bp (63.8bp)
2	UGAAGAAGAU	1e-626	-1.444e+03	56.65%	35.82%	49.3bp (71.5bp)
3	GAUGACUUUG	1e-320	-7.373e+02	39.67%	25.82%	53.5bp (67.2bp)
4	CAGCAGCAGC	1e-244	-5.628e+02	20.46%	11.30%	53.1bp (71.2bp)
5	UUUUCUACAG	1e-234	-5.401e+02	30.14%	19.33%	52.1bp (66.2bp)

I3_5'UTR

Rank	Motif	P-value	log P-pvalue	% of Targets	% of Background	STD(Bg STD)
1	ACCGAAGAAG	1e-20	-4.763e+01	8.98%	1.91%	49.7bp (72.9bp)
2	AGUAUUGACA	1e-13	-3.217e+01	1.89%	0.06%	46.3bp (58.7bp)
3	AACGGUAAGU	1e-13	-3.186e+01	7.40%	1.94%	48.7bp (66.2bp)
4	ACUUGUUUCU	1e-13	-3.026e+01	2.20%	0.12%	46.6bp (58.4bp)

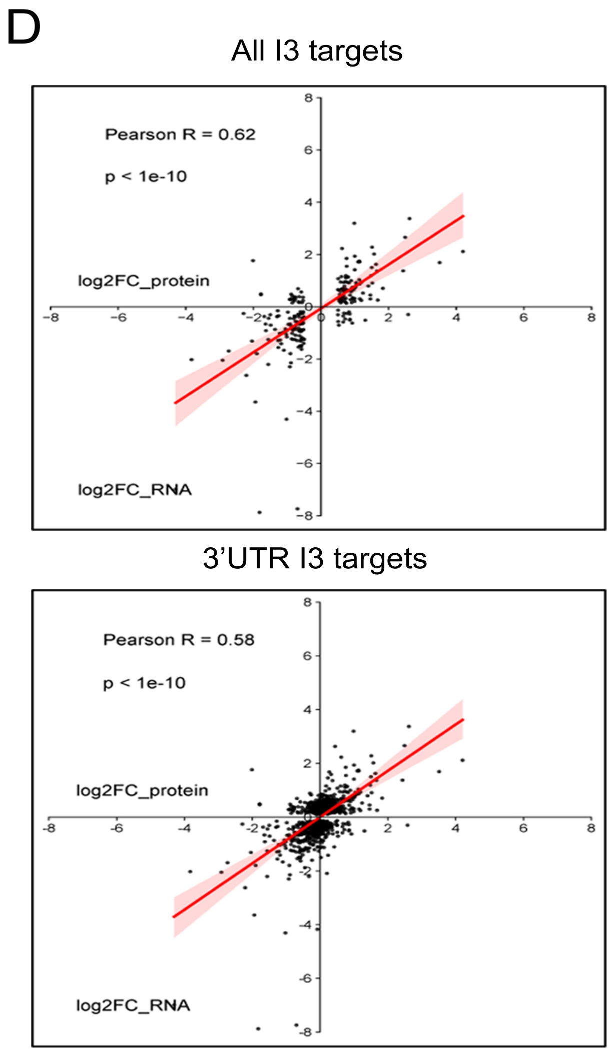

E

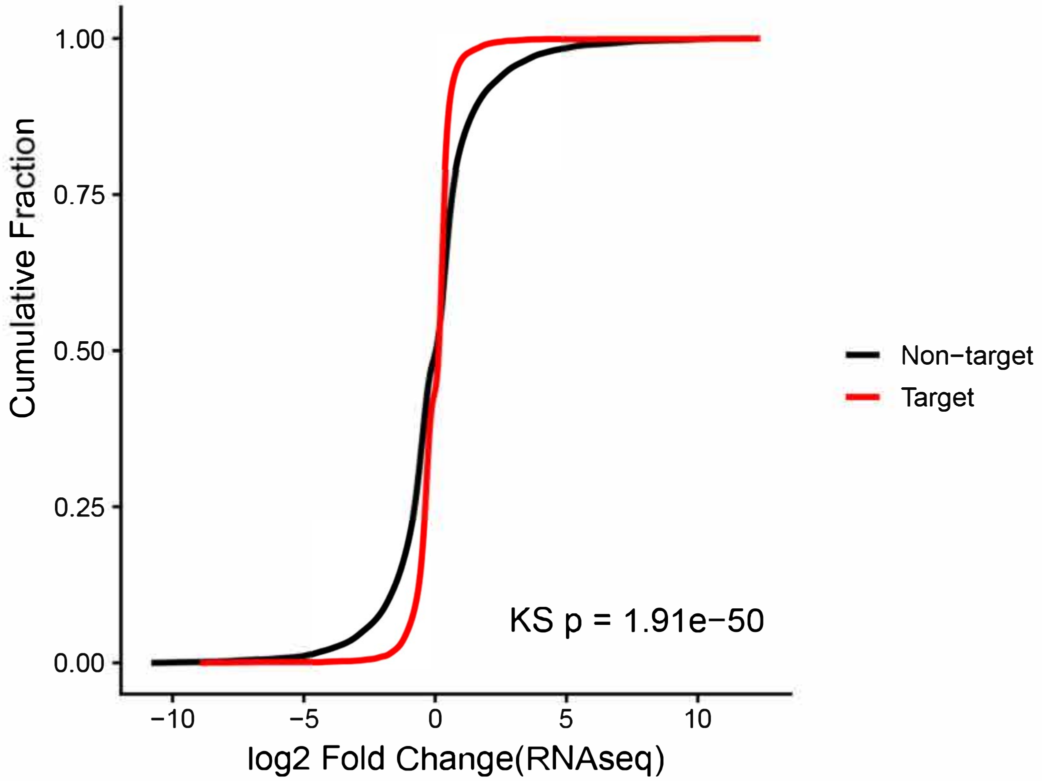

F

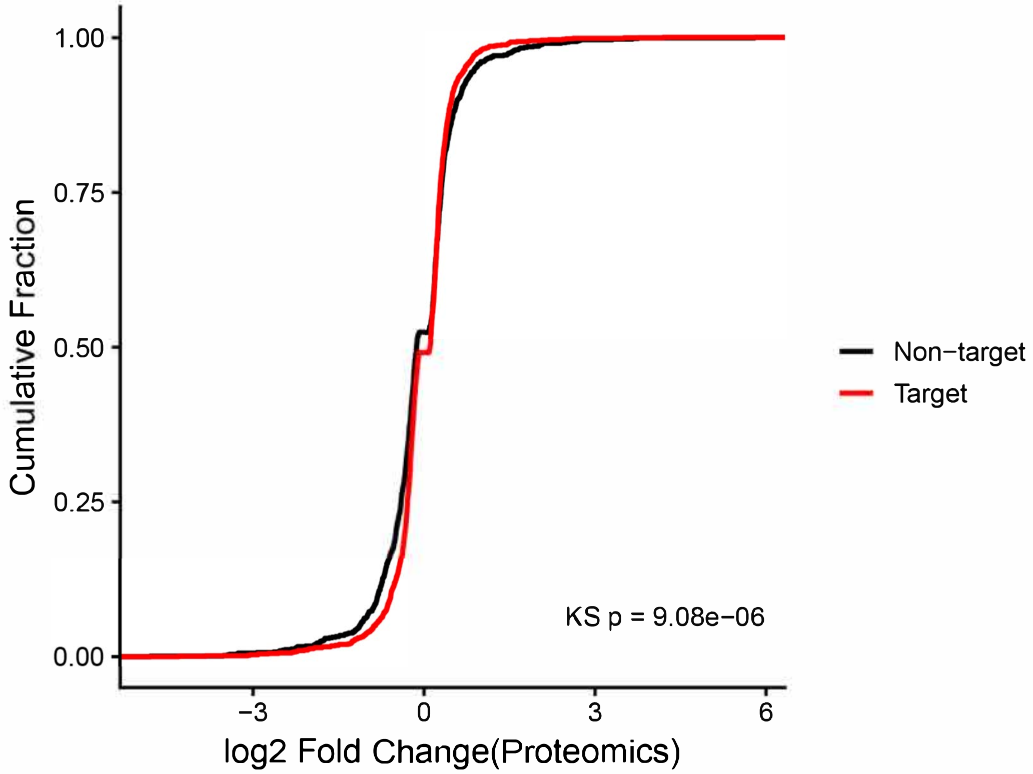

Supplementary figure 1. A. Schematic overview of the cell lines and genomic sequencing data sets produced for this study. **B.** Top: Western blots of control (NT) and I3-depleted (I3KO) SEM cells using an antibody against I3. GAPDH was used as a loading control. Bottom: Western blots of control (NT) SEM cells during I3 eCLIP using an antibody against I3. 10% of IP samples and 1% of input samples were loaded. **C.**Sequence motifs enriched in I3 eCLIP peaks across 3′UTR, CDS, and 5′UTR regions in NT cells. **D.** Scatter plots highlighting the positive correlation between RNA and protein for all I3 targets (top) and 3'UTR I3 targets (bottom) with a significant pearson correlation score. **E.** Cumulative distribution plot of RNA log2 fold change for I3 targets and non targets. **F**. Same as E but for protein log2 fold change.

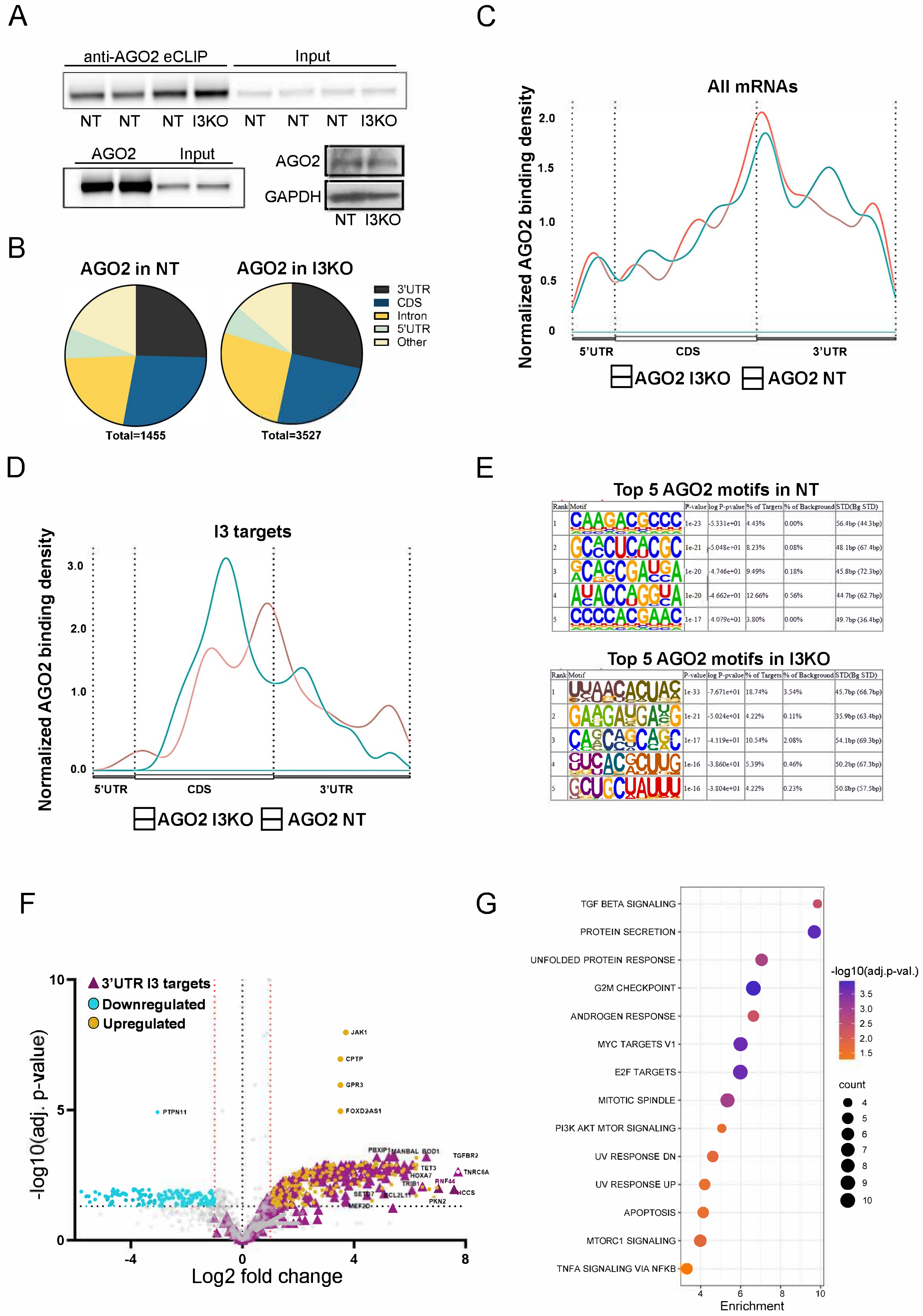

A
anti-AGO2 eCLIP
Input
NT NT NT I3KO NT NT NT I3KO
AGO2
Input
AGO2
GAPDH
NT I3KO
B
AGO2 in NT
AGO2 in I3KO
3'UTR
CDS
Intron
5'UTR
Other
Total=1455
Total=3527
C
All mRNAs
Normalized AGO2 binding density
2.0
1.5
1.0
0.5
0
5'UTR
CDS
3'UTR
AGO2 I3KO
AGO2 NT
D
I3 targets
Normalized AGO2 binding density
3.0
2.0
1.0
0.0
5'UTR
CDS
3'UTR
AGO2 I3KO
AGO2 NT
E
Top 5 AGO2 motifs in NT
Rank Motif P-value log P-pvalue % of Targets % of Background STD(Bg STD)
1 CAAGACGCCC 1e-23 -5.331e+01 4.43% 0.00% 56.4bp (44.3bp)
2 1e-21 -5.048e+01 8.23% 0.08% 48.1bp (67.4bp)
3 1e-20 -4.746e+01 9.49% 0.18% 45.8bp (72.3bp)
4 1e-20 -4.662e+01 12.66% 0.56% 44.7bp (62.7bp)
5 CCCCACGAAC 1e-17 -4.079e+01 3.80% 0.00% 49.7bp (36.4bp)
Top 5 AGO2 motifs in I3KO
Rank Motif P-value log P-pvalue % of Targets % of Background STD(Bg STD)
1 1e-33 -7.671e+01 18.74% 3.54% 45.7bp (66.7bp)
2 1e-21 -5.024e+01 4.22% 0.11% 35.9bp (63.4bp)
3 1e-17 -4.119e+01 10.54% 2.08% 54.1bp (69.3bp)
4 1e-16 -3.860e+01 5.39% 0.46% 50.2bp (67.3bp)
5 1e-16 -3.804e+01 4.22% 0.23% 50.8bp (57.5bp)
F
3'UTR I3 targets
Downregulated
Upregulated
-log10(adj. p-value)
10
5
0
-4
0
4
8
Log2 fold change
PTPN11
JAK1
CPTP
GPR3
FOXD2AS1
PBXIP1
MANBAL
BOD1
TGFBR2
TET3
TNRC6A
HOXA7
TRIB1
RNF44
SETD7
BCL2L11
HCCS
PKN2
MEF2D
G
TGF BETA SIGNALING
PROTEIN SECRETION
UNFOLDED PROTEIN RESPONSE
G2M CHECKPOINT
ANDROGEN RESPONSE
MYC TARGETS V1
E2F TARGETS
MITOTIC SPINDLE
PI3K AKT MTOR SIGNALING
UV RESPONSE DN
UV RESPONSE UP
APOPTOSIS
MTORC1 SIGNALING
TNFA SIGNALING VIA NFKB
-log10(adj.p-val.)
3.5
3.0
2.5
2.0
1.5
count
4
5
6
7
8
9
10
4
6
8
10
Enrichment

H

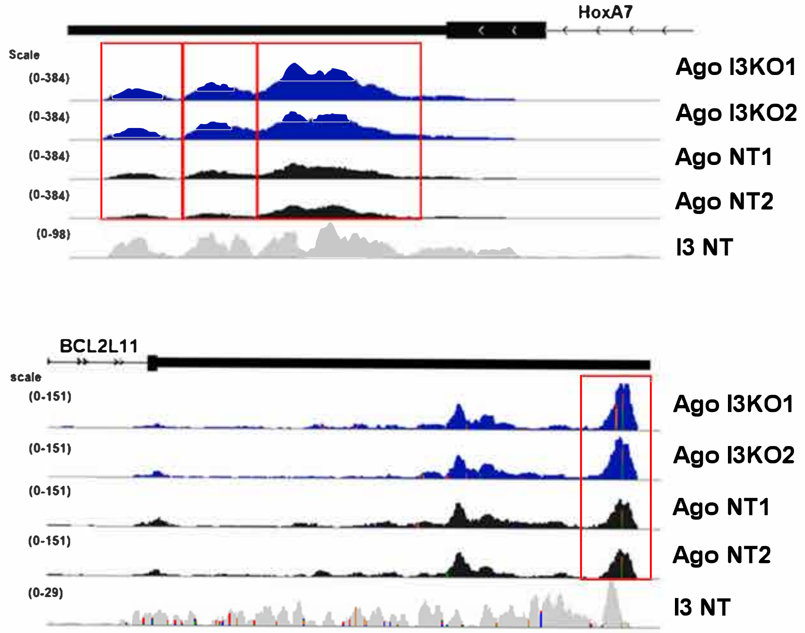

Supplementary figure 2. A. Western blots of control (NT) and I3-depleted (I3KO) SEM cells during AGO2 miR-eCLIP using an antibody against AGO2. 10% of IP samples and 1% of input samples were loaded and samples were run on two gels. Bottom right: Western blots of control (NT) and I3-depleted (I3KO) SEM cells using an antibody against AGO2. **B.** Distribution of AGO2 eCLIP peaks across gene features in control (NT) and I3 knock-out (I3KO) SEM cells. Total number of peaks are written out for each condition underneath the respective pie chart. **C.** Metagene plot depicting the relative density of AGO2 eCLIP peaks across gene features of all mRNAs in control (NT, blue) and I3-depleted (I3KO, red) SEM cells. Peak counts were normalized as counts per million relative to gene length. Same as C but with I3-bound mRNAs only. **E.** Top five HOMER binding motifs of AGO2 eCLIP peaks in control (NT) and I3-depleted SEM cells (I3KO). **F.** Volcano plot showing differential AGO2 eCLIP peaks between control (NT) and I3-depleted (I3KO) cells, with respect to I3KO. Significantly different eCLIP peaks are depicted as colored dots. AGO2 eCLIP peaks in 3'-UTRs also bound by I3 in NT are marked as purple triangles. The threshold for differential peaks was defined as less than -1 Log2 fold-change or greater than 1 Log2 fold-change, with the p-value <0.05. **G.** Hallmark enrichment analysis of mRNAs with increased 3'-UTR AGO2 eCLIP peaks in I3 knock-out cells (purple triangles from E). Significantly enriched pathways had an FDR of less than 0.05. **H.** IGV browser screenshots of HOXA7 and BCL2L11 3'-UTRs. AGO2 NT and I3KO eCLIP tracks are shown in black and blue respectively. I3 NT eCLIP tracks are shown in grey. Differential AGO2 peaks are highlighted with red boxes. The eCLIP peaks were normalized to input using bamcompare and the resulting normalized files were plotted on IGV.

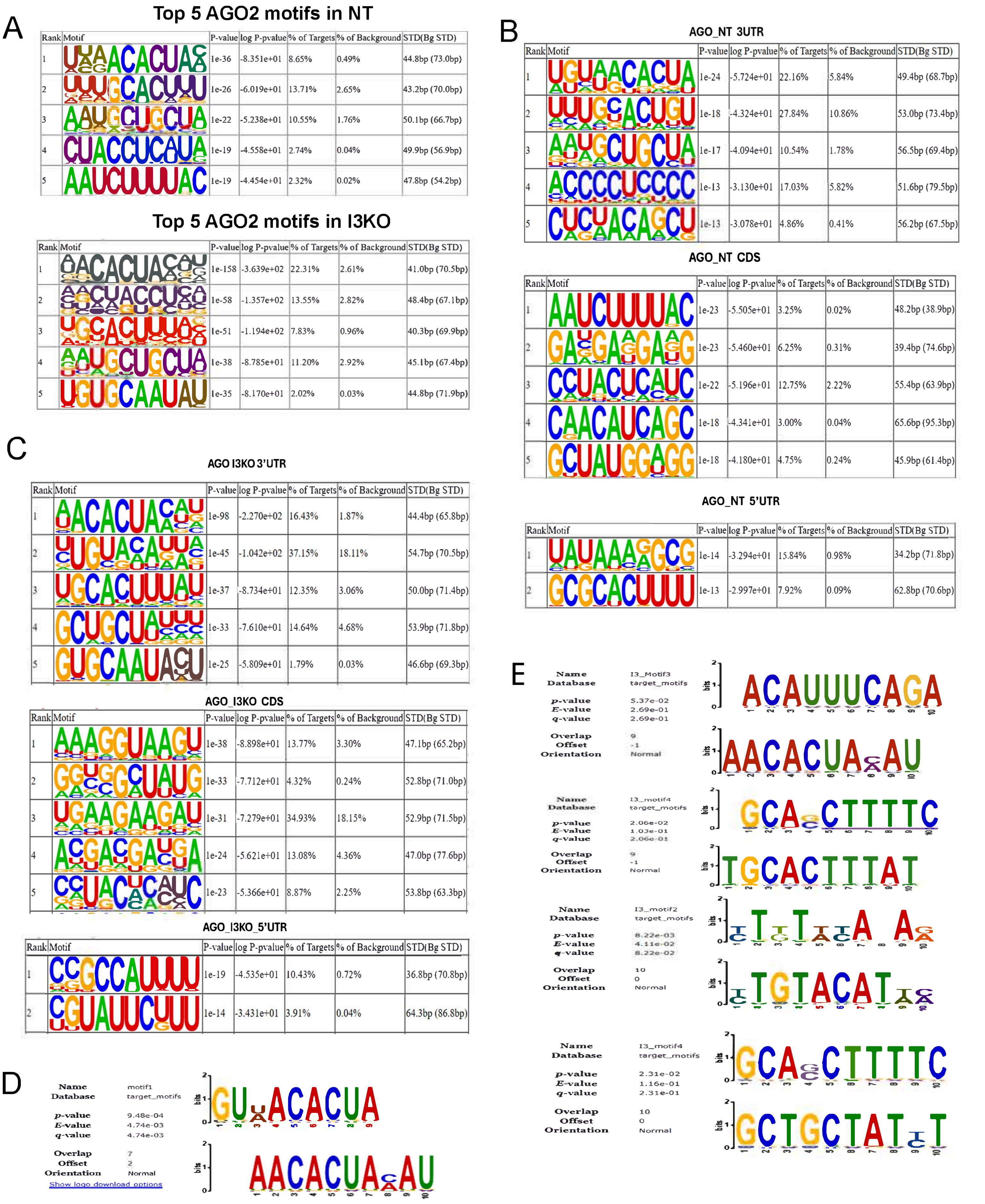

Rank	Motif	P-value	log P-pvalue	% of Targets	% of Background	STD(Bg STD)
1		1e-36	-8.351e+01	8.65%	0.49%	44.8bp (73.0bp)
2		1e-26	-6.019e+01	13.71%	2.65%	43.2bp (70.0bp)
3		1e-22	-5.238e+01	10.55%	1.76%	50.1bp (66.7bp)
4		1e-19	-4.558e+01	2.74%	0.04%	49.9bp (56.9bp)
5		1e-19	-4.454e+01	2.32%	0.02%	47.8bp (54.2bp)

Rank	Motif	P-value	log P-pvalue	% of Targets	% of Background	STD(Bg STD)
1		1e-158	-3.639e+02	22.31%	2.61%	41.0bp (70.5bp)
2		1e-58	-1.357e+02	13.55%	2.82%	48.4bp (67.1bp)
3		1e-51	-1.194e+02	7.83%	0.96%	40.3bp (69.9bp)
4		1e-38	-8.785e+01	11.20%	2.92%	45.1bp (67.4bp)
5		1e-35	-8.170e+01	2.02%	0.03%	44.8bp (71.9bp)

Rank	Motif	P-value	log P-pvalue	% of Targets	% of Background	STD(Bg STD)
1		1e-24	-5.724e+01	22.16%	5.84%	49.4bp (68.7bp)
2		1e-18	-4.324e+01	27.84%	10.86%	53.0bp (73.4bp)
3		1e-17	-4.094e+01	10.54%	1.78%	56.5bp (69.4bp)
4		1e-13	-3.130e+01	17.03%	5.82%	51.6bp (79.5bp)
5		1e-13	-3.078e+01	4.86%	0.41%	56.2bp (67.5bp)

Rank	Motif	P-value	log P-pvalue	% of Targets	% of Background	STD(Bg STD)
1		1e-23	-5.505e+01	3.25%	0.02%	48.2bp (38.9bp)
2		1e-23	-5.460e+01	6.25%	0.31%	39.4bp (74.6bp)
3		1e-22	-5.196e+01	12.75%	2.22%	55.4bp (63.9bp)
4		1e-18	-4.341e+01	3.00%	0.04%	65.6bp (95.3bp)
5		1e-18	-4.180e+01	4.75%	0.24%	45.9bp (61.4bp)

Rank	Motif	P-value	log P-pvalue	% of Targets	% of Background	STD(Bg STD)
1		1e-14	-3.294e+01	15.84%	0.98%	34.2bp (71.8bp)
2		1e-13	-2.997e+01	7.92%	0.09%	62.8bp (70.6bp)

Rank	Motif	P-value	log P-pvalue	% of Targets	% of Background	STD(Bg STD)
1		1e-98	-2.270e+02	16.43%	1.87%	44.4bp (65.8bp)
2		1e-45	-1.042e+02	37.15%	18.11%	54.7bp (70.5bp)
3		1e-37	-8.734e+01	12.35%	3.06%	50.0bp (71.4bp)
4		1e-33	-7.610e+01	14.64%	4.68%	53.9bp (71.8bp)
5		1e-25	-5.809e+01	1.79%	0.03%	46.6bp (69.3bp)

Rank	Motif	P-value	log P-pvalue	% of Targets	% of Background	STD(Bg STD)
1		1e-38	-8.898e+01	13.77%	3.30%	47.1bp (65.2bp)
2		1e-33	-7.712e+01	4.32%	0.24%	52.8bp (71.0bp)
3		1e-31	-7.279e+01	34.93%	18.15%	52.9bp (71.5bp)
4		1e-24	-5.621e+01	13.08%	4.36%	47.0bp (77.6bp)
5		1e-23	-5.366e+01	8.87%	2.25%	53.8bp (63.3bp)

Rank	Motif	P-value	log P-pvalue	% of Targets	% of Background	STD(Bg STD)
1		1e-19	-4.535e+01	10.43%	0.72%	36.8bp (70.8bp)
2		1e-14	-3.431e+01	3.91%	0.04%	64.3bp (86.8bp)

Supplementary figure 3. A. Top 5 AGo2 motifs in Nt (top) and I3KO(bottom) showing differences in motif diffrences in two conditions. **B-C.** AGO2 Motif analysis stratified across transcript features i.e. 3'UTR, CDS and 5'UTR in NT (B) and I3KO cells (C). **D.** Motif comparison between NT and I3KO resulted in one motif overlap for 3'UTR while other motifs showed no significant overlaps. **E.** Motif comparison between I3 and AGO I3KO motifs across 3'UTR regions reveal shared sequence features at sites where AGO2 binding increases in the absence of I3 .

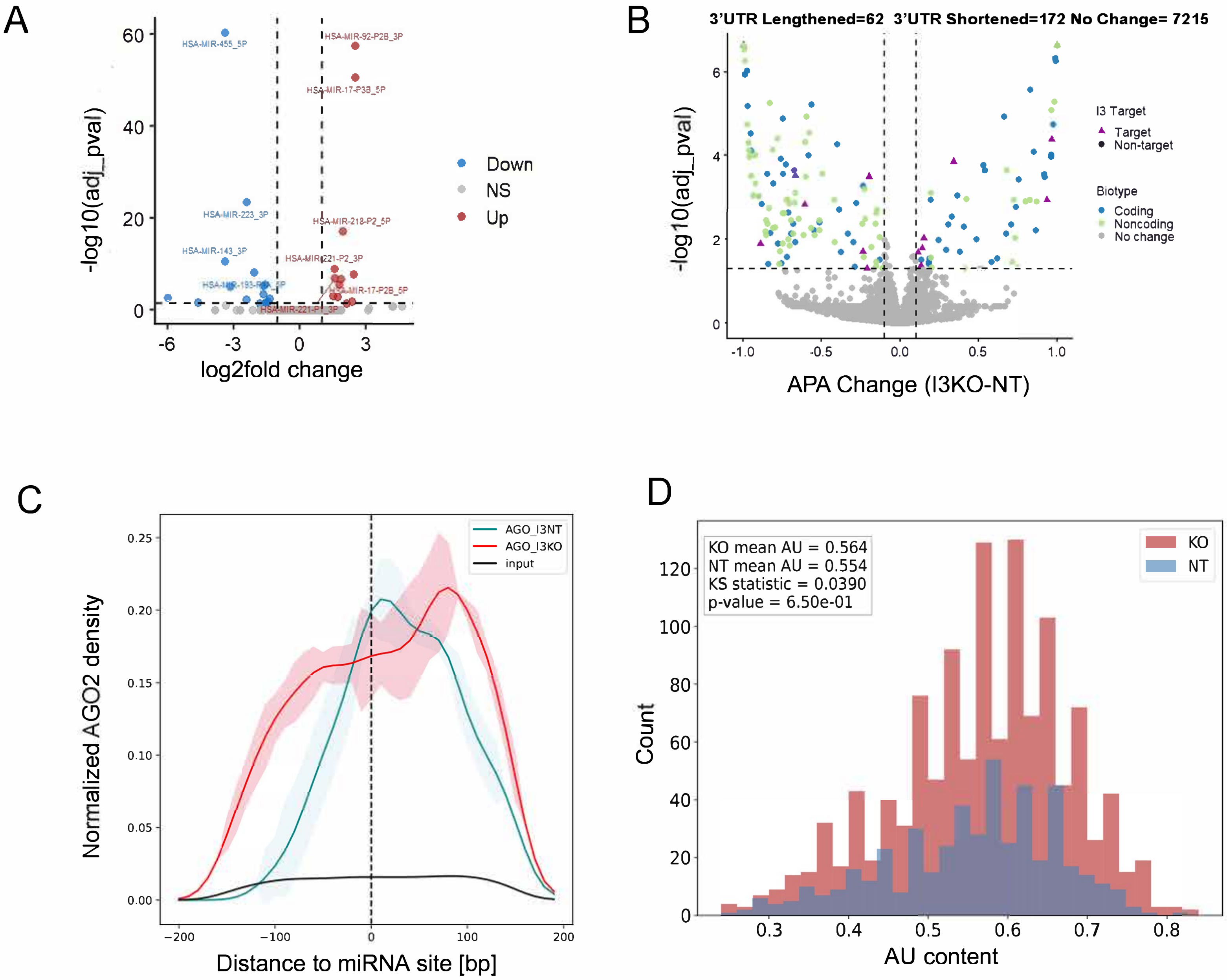

Supplementary figure 4. A. Volcano plot of small RNA□seq data showing significantly differentially expressed miRNAs between I3KO and NT cells where red(up) and blue(down) are labeled respectively. None of the miRNAs from miR-eCLIP data were signiifcantly expressed. **B.** Alternate Polyadenylation (APA) change in I3KO over NT cells. Volcano plot showing APA changes (I3KO/ NT) for all expressed transcripts. Vertical dashed lines mark thresholds for 3′UTR lengthening and shortening, and the horizontal dashed line indicates the adjusted P-value cutoff. Coding (blue), noncoding (green), and unchanged transcripts (gray) are shown alongside I3 eCLIP targets (purple triangles). A total of 62 transcripts lengthened, 172 shortened, and 7,215 showed no significant APA change. **C.** Metagene plot of AGO2 miR-eCLIP peaks in control (NT, blue) and I3 knock-out (I3KO, red) relative to annotated miRNA sites by miRBase. I3 eCLIP peaks in NT are depicted in grey. Peaks were normalized to CPM (counts per million) for all the replicates. **D.** AU-richness of AGO motif sequences in I3 NT and I3KO samples. Mean AU content was calculated for all motif-aligned sequences, and distributions were compared using a two-sample Kolmogorov–Smirnov test (KS test). Both conditions showed AU content below 0.6, with no significant difference in sequence composition or AU-richness between NT and KO.

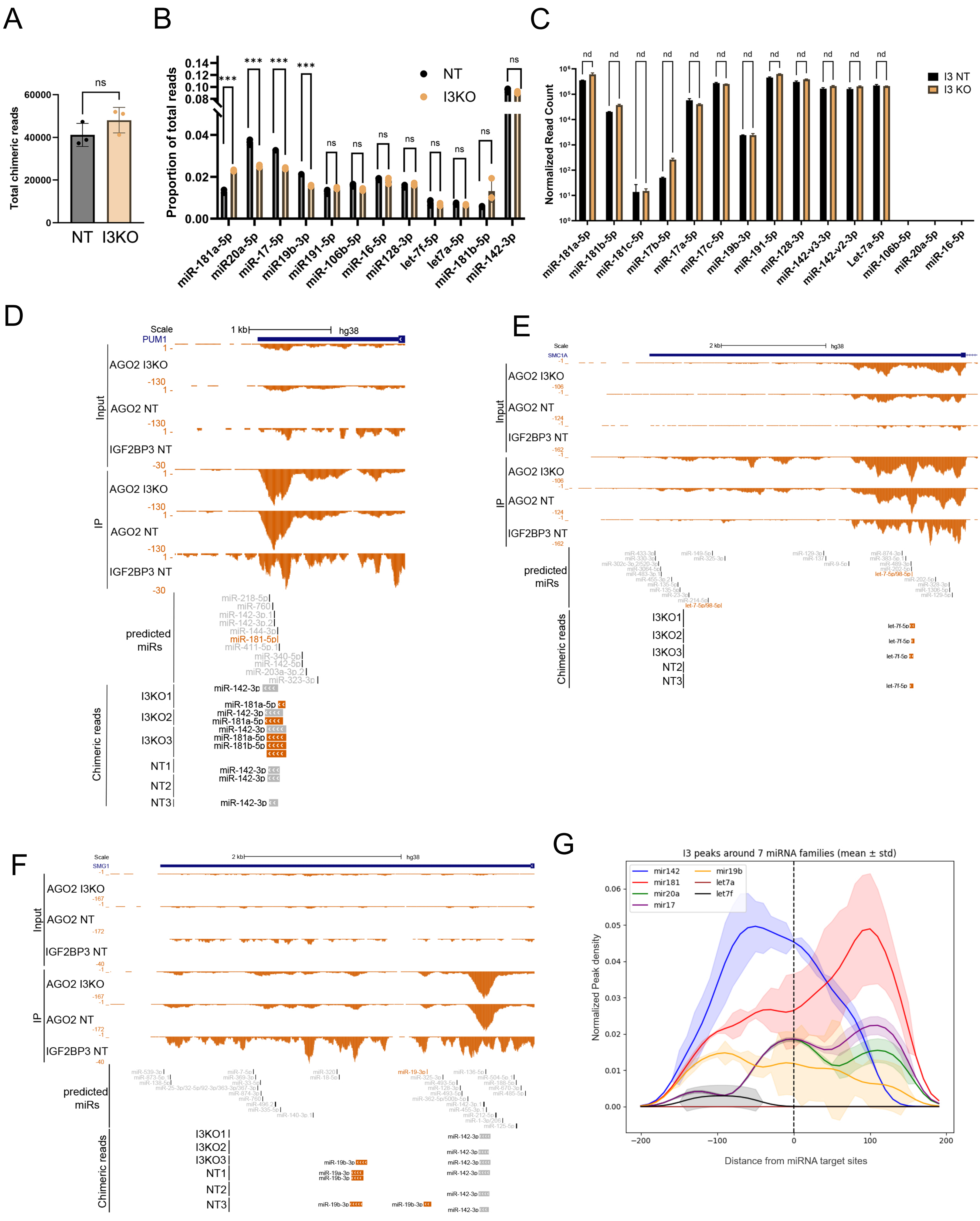

A
Total chimeric reads
ns
NT
I3KO
B
Proportion of total reads
NT
I3KO
miR-181a-5p
miR20a-5p
miR-17-5p
miR19b-3p
miR191-5p
miR-106b-5p
miR-16-5p
miR128-3p
let-7f-5p
let7a-5p
miR-181b-5p
miR-142-3p
C
Normalized Read Count
I3 NT
I3 KO
nd
D
Scale
1 kb
hg38
PUM1
Input
IP
AGO2 I3KO
AGO2 NT
IGF2BP3 NT
predicted miRs
Chimeric reads
I3KO1
I3KO2
I3KO3
NT1
NT2
NT3
E
SMC1A
2 kb
F
SMG1
G
I3 peaks around 7 miRNA families (mean ± std)
mir142
mir181
mir20a
mir17
mir19b
let7a
let7f
Normalized Peak density
Distance from miRNA target sites

H

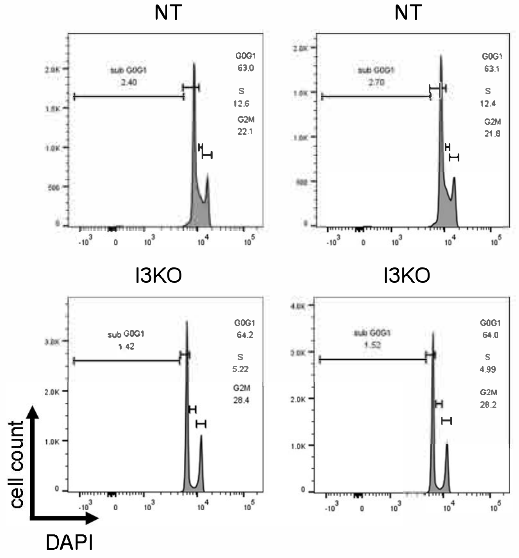

I

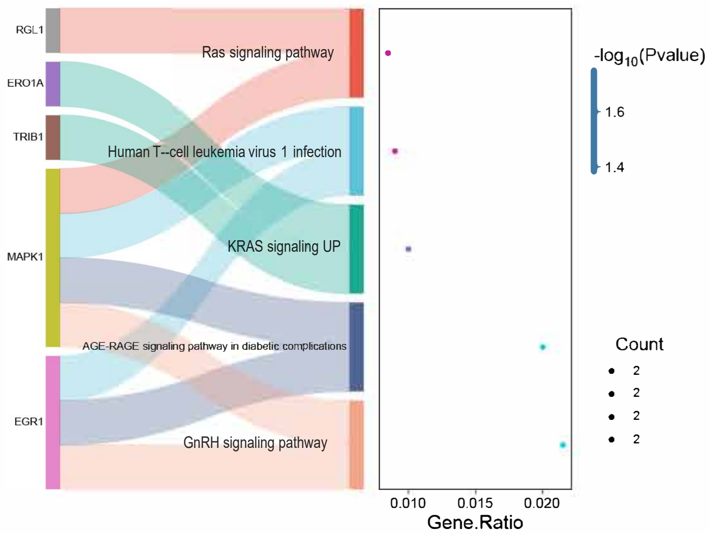

Supplementary figure 5. A. Chimeric read count proportions of most abundant miRNAs mapping to 3'-UTRs from AGO2 miR-eCLIP reads in control (NT, black) and I3 knock-out (I3KO, orange) cells. Chimeric read counts for each miRNA were normalized to total chimeric read count in each condition, yielding read proportions. Two-way ANOVA was used to calculate statistical significance. **B.** Quantification of total AGO2 miR-eCLIP chimeric reads across replicates in control (NT) and I3 knock-out (I3KO) SEM cells. An unpaired t-test was used to calculate statistical significance using the GraphPad Prism software. **C.** Normalized read counts of miRNAs identified by small RNA-seq in the control (NT, black) and I3 knock-out (I3KO, orange) cell lines. Two-way ANOVA was used to calculate the statistical significance. **D.** UCSC genome browser screenshot showing AGO2 chimeric eCLIP peaks on the 3'-UTR of the I3-dependent miR-181 target PUM1. Predicted microRNA binding sites (TargetScanHuman) are shown as a reference. Only microRNAs with predicted binding are shown in the chimeric read tracks. Non-chimeric AGO2 coverage tracks in control and I3 knock-out (I3KO), as well as I3 coverage tracks in control are shown as a reference. Size-matched inputs for each immunoprecipitation coverage track are also shown. **E**. Same as D. but showing the SMCA1 3'-UTR with I3-dependent let-7f binding. **F.** Same as D but showing the SMG1 3'-UTR with I3-dependent miR-19b binding. **G.** Metagene plot highlighting I3 occupancy across different miR targets. **H.** Corresponding FACS plots to figure 4B showing DAPI-stained SEM NT and I3KO cells. **I.** KEGG pathway enrichment analysis of mRNAs with decreased Differential Chimeric Read Occupancy (DCRO) from figure 3A.

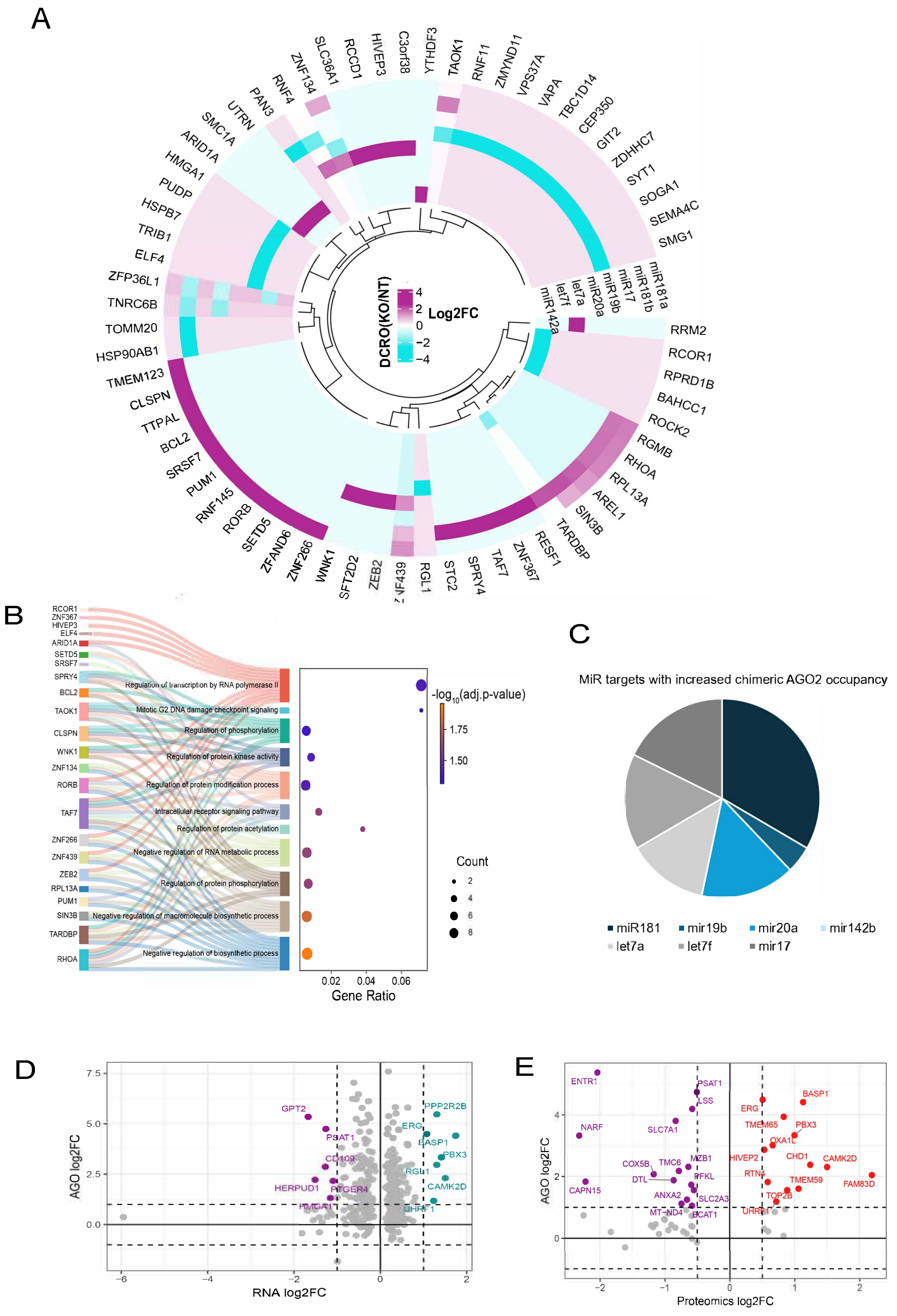

A
DCRO(KO/NT)
Log2FC
4
2
0
−2
−4
miR181a
miR181b
miR17
miR19b
miR20a
let7a
let7f
miR142a
RRM2
RCOR1
RPRD1B
BAHCC1
ROCK2
RGMB
RHOA
RPL13A
AREL1
SIN3B
TARDBP
RESF1
ZNF367
TAF7
SPRY4
STC2
RGL1
ZEB2
SFT2D2
WNK1
ZNF266
ZFAND6
SETD5
RORB
RNF145
PUM1
SRSF7
BCL2
TTPAL
CLSPN
TMEM123
HSP90AB1
TOMM20
TNRC6B
ZFP36L1
ELF4
TRIB1
HSPB7
PUDP
HMGA1
ARID1A
SMC1A
UTRN
PAN3
RNF4
ZNF134
SLC36A1
RCCD1
HIVEP3
C3orf38
YTHDF3
TAOK1
RNF11
ZMYND11
VPS37A
VAPA
TBC1D14
CEP350
GIT2
ZDHHC7
SYT1
SOGA1
SEMA4C
SMG1
B
RCOR1
ZNF367
HIVEP3
ELF4
ARID1A
SETD5
SRSF7
SPRY4
BCL2
TAOK1
CLSPN
WNK1
ZNF134
RORB
TAF7
ZNF266
ZNF439
ZEB2
RPL13A
PUM1
SIN3B
TARDBP
RHOA
Regulation of transcription by RNA polymerase II
Mitotic G2 DNA damage checkpoint signaling
Regulation of phosphorylation
Regulation of protein kinase activity
Regulation of protein modification process
Intracellular receptor signaling pathway
Regulation of protein acetylation
Negative regulation of RNA metabolic process
Regulation of protein phosphorylation
Negative regulation of macromolecule biosynthetic process
Negative regulation of biosynthetic process
-log10(adj.p-value)
1.75
1.50
Count
2
4
6
8
0.02
0.04
0.06
Gene Ratio
C
MiR targets with increased chimeric AGO2 occupancy
miR181
mir19b
mir20a
mir142b
let7a
let7f
mir17
D
7.5
5.0
2.5
0.0
AGO log2FC
−6
−4
−2
0
2
RNA log2FC
GPT2
PSAT1
CD109
HERPUD1
PTGER4
HMGA1
PPP2R2B
ERG
BASP1
PBX3
RGL1
CAMK2D
UHRF1
E
AGO log2FC
Proteomics log2FC
4
2
0
−2
−1
0
1
2
ENTR1
NARF
CAPN15
PSAT1
LSS
SLC7A1
COX5B
TMC6
DTL
MZB1
PFKL
ANXA2
SLC2A3
MT−ND4
BCAT1
BASP1
ERG
TMEM65
PBX3
OXA1L
HIVEP2
CHD1
CAMK2D
RTN4
TMEM59
FAM83D
TOP2B
UHRF1

Supplementary figure 6. A. Circle plot depicting Differential Chimeric Read Occupancy (DCRO) of miR-181 a/b, miR-17, miR-19b, miR-20, let-7a/f and miR-142 on 13 target mRNAs, between control (NT) and 13 knock-out (I3KO) cells, with respect to I3KO with a log2FC of +1 and -1 and p value of 0.05. **B** Hallmark enrichment analysis of mRNAs with increased Differential Chimeric Read Occupancy (DCRO) from panel A. Significantly enriched pathways had a FDR of less than 0.05. Genes part of the enriched pathways are annotated and connected by a Sankey plot. **C**. Pie chart showing the distribution of miRNA families whose targets exhibit increased AGO2 occupancy among IGF2BP3 bound transcripts, highlighting the dominant contribution of miR-181 relative to other miRNAs. miR142 has no overlaps with the AGo2 occupied I3 targets. **D-E.**Impact on RNA and protein levels of genes targeted by RISC. Scatter plot of AGO2 eclip Log2 fold changes with RNA **(D)** and protein (**E)** fold changes.

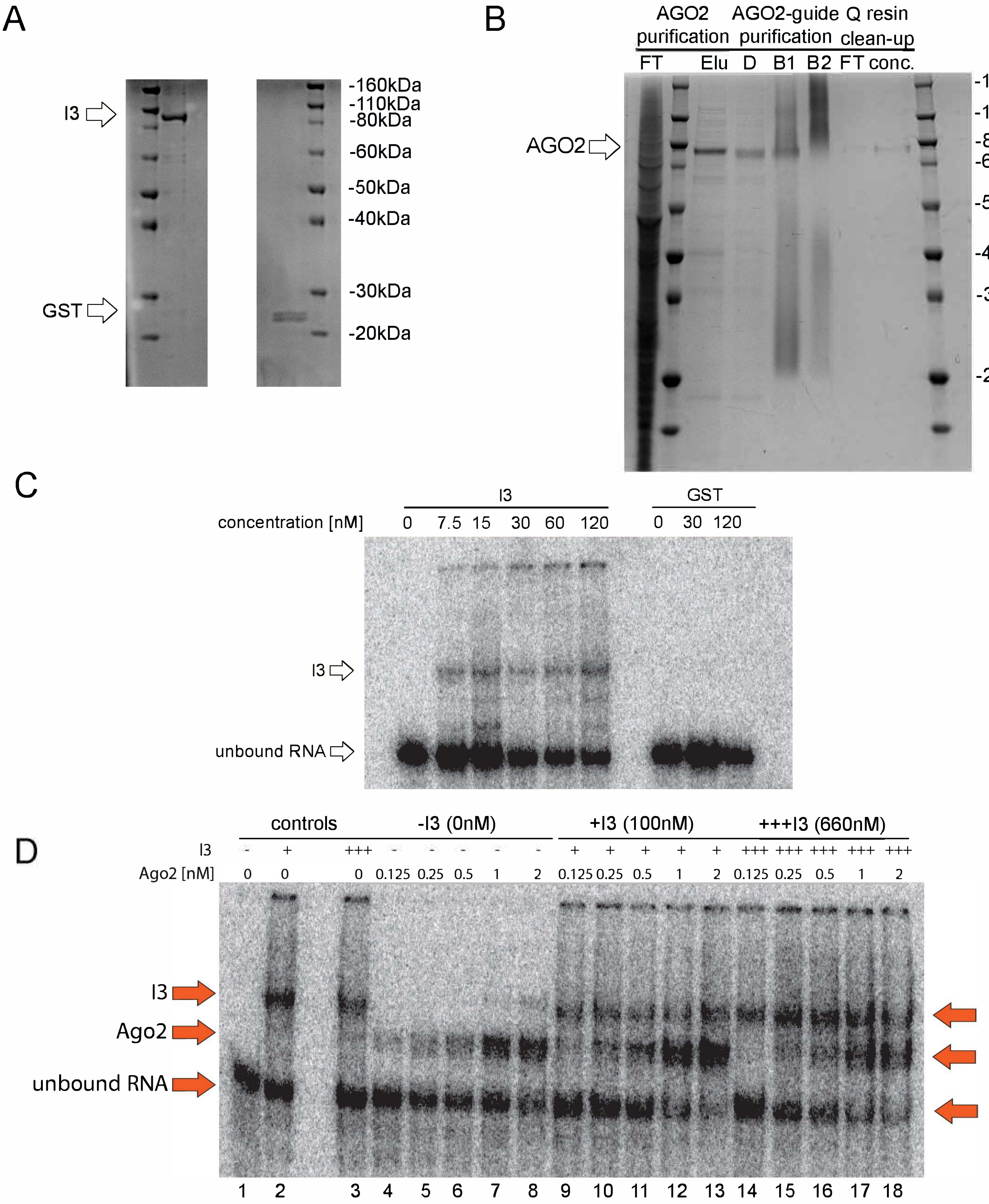

Supplementary figure 7. A. Coomassie-stained gel of recombinantly expressed and purified I3 after FPLC purification to exclude endogenous GST. **B.** Coomassie-stained gel of recombinantly expressed and purified AGO2 across the microRNA loading process. FT = flow-through (unbound proteins); Elu = elution (eluted AGO); D = purified AGO after microRNA loading; B1 = bead1 (bound AGO2-let7 to capture resin), B2 = bead2 (capture resin after AGO2-let-7 elution); FT = flow-through during Q-resin clean-up; conc. = concentrated and purified AGO2-let7 complex. **C.** Electrophoretic Mobility Shift Assay (EMSA) using recombinant I3-GST and GST. Partial 3'-UTR sequence from the oncogene HMGA2 was used as the RNA substrate. **D.** EMSA using recombinant I3 and AGO2 loaded with synthetic let-7a microRNA. The partial HMGA2 3'-UTR sequence carrying mutations at I3 binding sites was used as the RNA substrate. Lane 1 = no protein control, Lane 2 = sub-saturating (100nM) I3 only, Lane 3 = saturating (660nM) I3 only, Lane 4-7 = AGO2-let-7a titration without I3, Lane 8-12 =AGO2-let-7a titration with sub-saturating I3, Lane 13-18 = AGO2-let-7a titration with saturating I3.
