# Supplementary material for: IGF2BP3 remodels RISC occupancy to control microRNA targeting in leukemia
Source: NAR Cancer. 2026 Sep 23;8(3):zcag025. doi: 10.1093/narcan/zcag025 (PMC13598244; doi:10.1093/narcan/zcag025)
Supplement: Supplementary file 1 [file zcag025_supplemental_files.zip › Supplemental Methods.pdf]

# Supplemental Methods

## AU richness analysis

Sequence composition analysis was performed to quantify the nucleotide environment surrounding I3 motif instances. For each motif occurrence, the local sequence window was extracted and the fraction of A and U nucleotides was computed. AU content was calculated as (A+U)/(A+U+G+C) for each motif instance, and distributions were summarized across biological replicates for NT and KO samples. Mean AU content and confidence intervals were reported to evaluate differences in motif context between conditions.

## Alternative polyadenylation analysis for 3’ isoform usage

Alternative polyadenylation was quantified using DaPars2 (31, 32). Coordinate-sorted, indexed BAM files from I3 KO and NT RNA-seq samples were converted to per-base coverage wiggle files using bedtools genomecov with split-read handling. A RefSeq 3′UTR annotation and gene-mapping file was generated from hg38 reference annotation. DaPars2 was run in multi-sample mode to jointly infer proximal poly(A) sites and compute PDUI values. Differential APA between AGO2 KO and NT was determined using ΔPDUI and DaPars2 significance thresholds.

## Human IGF2BP3 expression and purification

Recombinant IGF2BP3 tagged with GST was created using the Baculovirus protein expression system. Plasmid, Bacmid and P2 Baculovirus generation were carried out by Genscript. Subsequently, P2 virus was used to infect Sf9 insect cells. Sf9 cells were infected for 3 days at 27 ˚C and harvested by centrifugation. Cell pellets were resuspended in lysis buffer (20mM

Tris-HCl pH 7.8, 100mM KCl, 0.2mM EDTA, 1mM DTT, 20% glycerol) and lysed in two passages through an Emulsiflex homogenizer (Avestin). Lysate was sonicated, clarified by centrifugation and tagged proteins were purified from the supernatant by incubating with Glutathione Sepharose 4B resin (Cytiva) for 20 minutes at 4°C. Resin was washed with Lysis Buffer and flow-through was continuously assessed for presence of contaminating protein by Bradford assay. After confirming that no more non-specific protein was present in the washes, IGF2BP3 was eluted off the resin using elution buffer (20mM Tris-HCl pH 7.8, 100mM KCl, 0.2mM EDTA, 1mM DTT, 20% glycerol, 10mM L-Glutathione). Elution was continuously assessed for presence of IGF2BP3 by Bradford until fully eluted from the resin. Eluted proteins were concentrated using a 50 kDa membrane Millipore concentrator. Subsequently, they were purified using size exclusion chromatography on a Superdex 200 Increase 10/300 GL column (Cytiva) on an Äkta Pure FPLC (Cytiva) using 20mM Tris-HCl pH 8, 100mM KCl, 0.2mM EDTA, 1mM DTT and 20% Glycerol. Protein concentration was determined by Bradford. Single-use aliquots were flash-frozen and stored at −80 °C until use.

## Preparation of human AGO2 loaded with synthetic miRNA

AGO2 loading with synthetic miRNA was combined with affinity purification and performed according to a previously published protocol with modifications (Gebert et al. 2021). Sf9 cells were infected for 72 hours at 27˚C and harvested by centrifugation. Pelleted cells were resuspended in Lysis buffer (50mM $NaH_2PO_4$ pH 8, 0.3M NaCl, and 0.5mM TCEP) and lysed by two passages through an Emulsiflex homogenizer (Avestin). The lysate was clarified by centrifugation and Nickel affinity purification of AGO2 was performed using Ni Sepharose excel resin (Cytiva) for 1.5 hours at 4°C. Non-specific proteins were removed by washing the resin twice with 10 column volumes of wash buffer (50mM Tris pH 8.0, 0.3M NaCl, 15mM Imidazole, 0.5mM TCEP). Subsequently, the resin was washed with 10 column volumes of Calcium wash

buffer (50mM Tris pH 8.0, 0.3M NaCl, 15mM Imidazole, 0.5mM TCEP, 5mM CaCl2). After the washes, a micrococcal nuclease treatment was performed to remove residual RNA. To that end, the resin was resuspended in Calcium Wash buffer and a micrococcal nuclease (Takara) was added. The resin was incubated for 40 minutes at room temperature with gentle inversion 2-3 times throughout the incubation period. After the digest, the resin was washed twice with 10 column volumes of Wash Buffer. AGO2 was eluted from the resin by resuspending it in 2 column volumes of Elution Buffer and incubating it on ice for 5 minutes. Eluted AGO2 was collected through gravity flow on a polypropylene column. The resin was resuspended once more in 1 column volume of the Elution buffer and flow-through was collected. EGTA was added to the purified AGO2 eluate to a final concentration of 5mM. Synthetic miRNA (Genscript) was then added to the eluate and incubated for approximately 5 minutes at 4°C. The resulting AGO2-miRNA mixture was dialyzed overnight at 4°C into Wash Buffer using a Slide-A-Lyzer 10kDa dialysis cassette (Thermo Scientific). Subsequently, AGO2 molecules loaded with the synthetic miRNA were purified and isolated from those bound to co-purifying cellular small RNAs as described previously (Flores-Jasso et al. 2013; Gebert et al. 2021) . Briefly, AGO2 loaded with a specific miRNA was captured using an antisense oligonucleotide (GenScript) and eluted with a DNA competitor (GenScript). Finally, the protein was dialyzed sequentially in Q Dialysis buffer (30mM Tris pH 8.0, 0.15M NaCl, 0.02% CHAPS, 0.5mM TCEP) and passed through Q Sepharose Fast Flow resin (Cytiva) to remove excess nucleic acids. Protein concentration was determined using Bradford. Single-use aliquots were flash frozen and stored at −80 °C until use.

The bacterial expression construct for full-length 6xHis-tagged human AGO2 protein was obtained from the lab of Professor Ian McRae (Scripps Research, San Diego, USA). A list of the used oligonucleotides can be found in Table 2.

References:


1. Flores-Jasso CF, Salomon WE, Zamore PD. 2013. Rapid and specific purification of Argonaute-small RNA complexes from crude cell lysates. RNA. 19(2):271–279. https://doi.org/10.1261/rna.036921.112
2. Gebert LFR, Law M, MacRae IJ. 2021. A structured RNA motif locks Argonaute2:miR-122 onto the 5’ end of the HCV genome. Nat Commun. 12(1):6836. https://doi.org/10.1038/s41467-021-27177-9
3. Feng,X., Li,L., Wagner,E.J. and Li,W. (2018) TC3A: The Cancer 3′ UTR Atlas. *Nucleic Acid Res.*, **46**, D1027–D1030.
4. Li,L., Huang,K.-L., Gao,Y., Cui,Y., Wang,G., Elrod,N.D., Li,Y., Chen,Y.E., Ji,P., Peng,F., *et al.* (2021) An atlas of alternative polyadenylation quantitative trait loci contributing to complex trait and disease heritability. *Nat. Genet.*, **53**, 994–1005.
